# Comparative evaluation of antimicrobial peptides: effect on formation, metabolic activity and viability of *Klebsiella pneumoniae* biofilms

**DOI:** 10.3389/fmicb.2025.1548362

**Published:** 2025-04-11

**Authors:** Sophia Hanstein, Thomas Grochow, Marina Mötzing, Simone A. Fietz, Ralf Hoffmann, Christoph G. Baums, Sophie Kähl

**Affiliations:** ^1^Institute of Bacteriology and Mycology, Center for Infectious Diseases, Faculty of Veterinary Medicine, Leipzig University, Leipzig, Germany; ^2^Institute of Veterinary Anatomy, Histology and Embryology, Faculty of Veterinary Medicine, Leipzig University, Leipzig, Germany; ^3^Institute of Bioanalytical Chemistry, Faculty of Chemistry, Leipzig University, Leipzig, Germany; ^4^Center for Biotechnology and Biomedicine, Leipzig University, Leipzig, Germany

**Keywords:** *Klebsiella pneumoniae*, biofilm formation, LL-37, human beta-defensin-3, proline-rich AMPs, DJK-5

## Abstract

**Introduction:**

*Klebsiella pneumoniae (K. pneumoniae)* is a major human nosocomial infectious agent and an important veterinary pathogen, frequently resistant to various antibiotics. It causes diseases such as pneumonia, urinary tract infections, surgical wound infections and septicemia. Biofilm formation of *K. pneumoniae* promotes persistent infection and contributes to resistance against antimicrobial agents. The objective of this study was to comparatively evaluate the effect of selected AMPs on the formation, metabolic activity and viability of *Klebsiella pneumoniae* biofilms of veterinary and human origin.

**Methods:**

Biofilm formation of three *K. pneumoniae* strains was quantified using the crystal violet assay and visualized by scanning electron microscopy (SEM). The inhibitory effects of eight different AMPs on the formation and metabolic activity of *K. pneumoniae* biofilms, as well as on planktonic growth, were examined using crystal violet, resazurin and broth microdilution assays, respectively. The effect on living and dead bacteria in mature biofilms was investigated using the fluorescent dyes SYTO™ 9 and propidium iodide. In addition, the distribution of rhodamine B-labeled peptide DJK-5 in mature biofilms of strain 17349 was visualized by confocal laser scanning microscopy (CLSM).

**Results:**

Biofilm formation was confirmed for all three *K. pneumoniae* strains. Depending on the strain, we found that planktonic growth was affected by the AMPs DJK-5, DJK-6, Onc72, and Onc112. Biofilm formation of all three strains was inhibited by hbD3, LL-37, DJK-5, and DJK-6, with biofilm mass reduced to less than 40% of the untreated control. In addition to the inhibition of biofilm formation, a reduction in the metabolic activity of the biofilm-associated bacteria was also observed. These four AMPs also showed an effect on mature biofilms by reducing the number of both viable and dead bacteria in 22 h-old biofilms. Rhodamine B-labeled DJK-5 took 7 h to visibly accumulate in the planktonic bacteria. Multi-layered biofilm aggregations were mainly negative for rhodamine B-labeled DJK-5, even 44 h after AMP treatment, indicating that certain parts of mature *K. pneumoniae* biofilms are not accessible for this AMP.

**Conclusion:**

In conclusion, we found differences in the effect of AMPs on biofilms including both increases and decreases in biofilm mass and viability.

## 1 Introduction

The term “ESKAPE” describes a small group of pathogens causing nosocomial infections, which are threatening because of increasing prevalence in hospitals and raising antimicrobial resistance (Rice, 2008). One of the pathogens belonging to this group is *Klebsiella pneumoniae* (*K. pneumoniae*), a Gram-negative, opportunistic pathogen of the family Enterobacteriaceae causing diseases in humans and animals (Podschun and Ullmann, 1998). In humans, *K. pneumoniae* colonizes the gastrointestinal tract and nasopharynx and is able to cause pneumonia, urinary tract infections, surgical wound infections, septicemia, endocarditis and cystitis (Rahmat Ullah et al., 2024). *K. pneumoniae* is also a common veterinary infectious agent causing pathologies such as septicemia in suckling piglets as well as mastitis, pneumonia, wound and genital-urinary tract infections in different animals (Quinn, 2011; Bowring et al., 2017; Bidewell et al., 2018; Ribeiro et al., 2022). Of particular concern is the resistance of many strains to various antibiotics due to the formation of extended-spectrum beta-lactamases (ESBLs) or carbapenemases (Rahmat Ullah et al., 2024). The WHO classified third-generation cephalosporin-resistant and carbapenem-resistant *K. pneumoniae* as a priority pathogen for which new antibiotics are urgently needed (WHO, 2024). Another critical trait of *K. pneumoniae* is the capability of forming biofilms, both inside the host and on medical devices, for example catheters and endotracheal tubes (Rahmat Ullah et al., 2024).

Biofilms are defined as an accumulation of bacteria embedded in an extracellular matrix consisting of polysaccharides, proteins, nucleic acids and lipids (Guerra et al., 2022). Resistance to antibiotic agents, e.g., ampicillin or ciprofloxacin, is increased in *K. pneumoniae* through biofilm formation (Anderl et al., 2003). It is estimated that 65–80% of all bacterial infections are biofilm-related and the threat of biofilm-forming pathogens is increasing for human health (Guerra et al., 2022; Barman et al., 2024).

Due to the current threat of multidrug-resistant *K. pneumoniae* and also biofilm-related infections new antimicrobials and especially biofilm targeting agents are urgently needed. Antimicrobial peptides (AMPs) are small, cationic, amphipathic peptides consisting of less than 60 amino acid residues (De Souza et al., 2022) and are considered a promising alternative to conventional antibiotics. The synthetic peptides 1018, DJK-5, and DJK-6 (Table 1) are described to inhibit biofilm formation and partly even eradicate mature biofilms of *K. pneumoniae* presumably by degradation or prevention of accumulation of the signalling nucleotides guanosine 5′-diphosphate 3′-diphosphate (ppGpp) and guanosine 5′-triphosphate 3′-diphosphate (pppGpp), which are induced by stress conditions and are involved in biofilm formation (La Fuente-Núñez et al., 2014; La Fuente-Núñez et al., 2015; Ribeiro et al., 2015). Peptide 1037 also has a described antibiofilm effect by reducing swimming motility and bacterial swarming and stimulating twitching motility (La Fuente-Núñez et al., 2012). An antibiofilm effect was also reported for the human beta-defensin-3 (hbD3) in *Staphylococcus* sp., *Pseudomonas aeruginosa* (*P. aeruginosa*) and multispecies biofilms (Lee et al., 2013; Zhu et al., 2013; Parducho et al., 2020) and for the human cathelicidin LL-37 in *P. aeruginosa, Escherichia coli (E. coli), Acinetobacter baumannii (A. baumannii), Staphylococcus epidermidis* (*S. epidermidis*), *Staphylococcus aureus* (*S. aureus*) and *K. pneumoniae* (Aka, 2015; Chen et al., 2018; Lin et al., 2018; Wongkaewkhiaw et al., 2020). HbD3 inhibits biofilm formation by influencing the expression of various biofilm-associated genes, for example *icaA*, *icaD*, and *icaR* (Zhu et al., 2013), while LL-37 affects bacterial biofilms through different mechanisms of action, for example the reduction of bacterial attachment to the surface, stimulation of twitching motility, quorum sensing pathway suppression and the up- and downregulation of certain biofilm-associated genes (e.g., associated with flagella or type IV pili) (Overhage et al., 2008; Dean et al., 2011; Demirci et al., 2022).

**Table 1:** Antimicrobial peptides used in this study.

AMP	Origin	Sequence	Purity	Net charge^a^	Molecular weight (g/mol)^a^	Hydrophilicity^a^
hbD3	Human defensin	GIINTLQKYYCRVRGGRCAVLSCLPKEEQIGKCSTRGRKCCRRKK	No data	11	5161.23	0.46
LL-37	Human cathelicidin	LLGDFFRKSKEKIGKEFKRIVQRIKDFLRNLVPRTES-OH	95.30%	6	4493.33	0.62
DJK-5	Synthetic	vqwrairvrvir-NH_2_	99%	5	1550.92	0.02
DJK-6	Synthetic	vqwrrirvwvir-NH_2_	99%	5	1666.06	- 0.23
1018	Synthetic	VRLIVAVRIWRR-NH_2_	94%	5	1535.95	- 0.15
1037	Synthetic	KRFRIRVRV-NH_2_	99%	6	1228.56	0.86
Onc72	Insect-derived	VDKPPYLPRPRPPROIYNO-NH_2_	95%	4	2077.46	0.32
Onc112	Insect-derived	VDKPPYLPRPRPPRrIYNr-NH_2_	87%	6	2389.84	0.32

a Values were calculated with Peptide Calculator (https://www.bachem.com/knowledge-center/peptide-calculator/).

The proline-rich AMPs (PrAMPs) Onc72 and Onc112 are derivates of the PrAMP Oncopeltus antibacterial peptide 4, which was originally isolated from the insect *Oncopeltus fasciatus* (Schneider and Dorn, 2001), the large milkweed bug (Knappe et al., 2010; Knappe et al., 2011a; Knappe et al., 2011b) and bind inside the tunnel of the 70S ribosome, block the peptidyl transferase center and block and destabilize the initiation complex (Krizsan et al., 2014; Roy et al., 2015; Seefeldt et al., 2015; Gagnon et al., 2016). Even though the antibacterial effect of Onc72 and Onc112 against various pathogens has been demonstrated *in vitro* and in mouse models (Knappe et al., 2012; Knappe et al., 2015; Kolano et al., 2020), no results are yet available on possible antibiofilm effects.

The aim of this study was to comparatively evaluate the effect of different AMPs on the formation, metabolic activity and viability of *Klebsiella pneumoniae* biofilms of veterinary and human origin. Three different *K. pneumoniae* strains, a pig isolate (17349), a dog and ESBL-producing isolate (IMT40061) and a human and hypervirulent isolate (42KC-108224-1) were investigated. AMPs with different modes of action were selected for this study, which previously were either part of investigations in our working group or described as possible biofilm affecting AMPs.

## 2 Materials and methods

### 2.1 Bacterial strains

Three different strains of *K. pneumoniae* were investigated in this study which were originally isolated from a human, a pig and a dog (Table 2) and characterized in previous studies (Krieger et al., 2021; Krieger et al., 2022). Minimum inhibitory concentration (MIC) values were determined according to EUCAST guidelines by microdilution test and are shown in Supplementary Table S1.

**Table 2:** *Klebsiella pneumoniae* strains used in this study.

Strain	Origin	Localization	Country	Year of isolation	Multi locus sequence type	Hypervirulence-associated factors						
						_p_rmpA	_p_rmpA2	_c_rmpA	iucA^d^	entB	ironB	irp2
17349^b^	Pig	Heart valve	Germany	2017	ST4741	−	−	−	+	+	−	+
42KC-108224-1^a^	Human	Bile	Germany	2016	ST65	+	+	−	+	+^c^	+^c^	+^c^
IMT40061^b^	Dog	Urine	Germany	2016	ST11	−	−	−	−	+	−	+

a Strain characterization by Krieger et al. (2021).

b Strain characterization by Krieger et al. (2022).

c Unpublished data.

d This gene was detected with two different primer pairs leading to the same result.

*Muribacter muris* (*M. muris*) strain 1040/11, a non-biofilm former (Sager et al., 2015), was used as a negative control for biofilm formation.

Frozen stocks of *K. pneumoniae* strains were prepared for indicated assays as follows. Bacteria were grown in brain-heart infusion (BHI) until late exponential phase (OD_600_ ~ 1,5), were mixed with glycerol to a final concentration of 15% and were subsequently frozen in liquid nitrogen and stored at −80°C until use.

### 2.2 Antimicrobial peptides

Peptides DJK-5, DJK-6, Onc72, Onc112, 1018, and 1037 (Table 1) were synthesized using the 9-fluorenylmethoxy-carbonyl*/tert*-butyl (Fmoc/^t^Bu) strategy with *in situ* DIC/HOBT activation (8 eq.) in DMF, and Fmoc-Rink amide AM resin (100–200 mesh, loading capacity, 0.62 mmol/g) on a multiple synthesizer (SYRO2000, MultiSynTech GmbH, Witten, Germany) as described previously (Knappe et al., 2010).

The Fmoc-group was cleaved with piperidine in DMF (40% v/v for 3 min and 20% v/v for 10 min). To obtain fluorophore-labeled peptides, rhodamine B was manually coupled to the N-terminus of DJK-5 upon completion of the peptide synthesis without linker. Peptides were cleaved with TFA containing 12.5% (v/v) of a scavenger mixture (1,2-ethandithiole, m-cresol, thioanisole, and water, 1:2:2:2, v/v/v/v) for 3 h, precipitated with cold diethyl ether, washed twice with diethyl ether, and dried. Crude peptides were purified on an Äkta Purifier 10 (GE Healthcare Europe GmbH, Freiburg, Germany) using a Jupiter C_18_-column (inner diameter: 10 mm, length: 250 mm, particle size: 5 μm, pore size: 300 Å; Phenomenex Inc., Torrance, USA). Eluent A was 0.1% (v/v) aqueous TFA and eluent B was 60% (v/v) aqueous acetonitrile containing 0.1% (v/v) TFA as ion pair reagent. Purifications relied on linear gradients with a slope of 1% acetonitrile per minute. RP-HPLC-MS was used to assess peptide purities. Samples were analyzed on an ACQUITY Arc system (Waters GmbH, Eschborn, Germany) coupled online to a detector recording the absorbance at 214 nm and an ion trap mass spectrometer equipped with an electrospray ionization source (ESI-IT-MS, Esquire HCT, Bruker Daltonics) operated in positive ion mode. Separations were performed on a Jupiter C_18_-column (inner diameter: 2 mm, length: 150 mm, particle size: 5 μm, pore size: 300 Å; Phenomenex) at a flow rate of 0.2 mL/min and a column temperature of 60°C using a linear gradient from 3 to 60% eluent D in 28.5 min. The eluents were 0.1% (v/v) aqueous formic acid (eluent C) and 100% (v/v) acetonitrile containing 0.1% (v/v) formic acid (eluent D). The ESI source was operated at a source temperature of 365°C using nitrogen curtain gas (40 psi) and dry gas (9 L/min). The purity of RhoB_DJK-5 was also checked by RP-HPLC using a Beckman System Gold HPLC (Beckman Coulter GmbH, Krefeld, Germany). Separation was performed on a Jupiter C18-column (inner diameter: 2 mm, length: 150 mm, particle size: 5 μm, pore size: 300 Å; Phenomenex) at a flow rate of 0.2 mL/min and a column temperature of 60°C using a linear gradient from 5 to 95% eluent B in 30 min. The absorbance was recorded at 214 nm. Purity of the antimicrobial peptides is shown in Table 1 and Supplementary Figure S1.

LL-37 was purchased from GENAXXON bioscience GmbH (P2299.9501, Ulm, Germany) and hbD3 was purchased from PeptaNova GmbH (SKU: 4382-s, Sandhausen, Germany). Stocks of antimicrobial peptide solution in sterile distilled water were prepared and stored at −20°C.

### 2.3 Determination of biofilm formation

#### 2.3.1 Crystal violet assay for quantification of biofilm formation

To quantify biofilm formation, the crystal violet assay was done as described previously (Sager et al., 2015). Specifically, bacteria were grown in 10 mL BHI broth for 18 h at 37°C on a shaking platform (120 rpm). Subsequently 100 μL of a 1:100 dilution of this overnight culture in BHI was transferred to 96-well plates and incubated for 24 h at 37°C. Wells with BHI broth only were used as blank. Five replicates were investigated per strain. After incubation the suspension was discarded, plates were washed three times with tap water and air-dried. Subsequently, 100 μL of 1% crystal violet (T123.1, Th. Geyer GmbH & Co. KG, Renningen, Germany) in distilled water was added to each well and incubated for 15 min at room temperature before rinsing the plates four times with tap water and air-drying them again. One hundred μL of 99% ethanol were added to each well for 15 min at room temperature to dissolve the remaining crystal violet. Subsequently, the optical density at 540 nm (OD_540_) was measured using the BioTek Synergy H1 microplate reader to quantify biofilm mass remaining in the wells. OD values of the blank were subtracted from all the samples. Each experiment was conducted three times.

To separate biofilm formers from non-biofilm formers, a cut-off value (ODc) was calculated as described in the literature (Stepanović et al., 2007), defined as the mean value of the negative control (*M. muris*) plus three times the standard deviation.

#### 2.3.2 Scanning electron microscopy for visualization of biofilm formation

To visualize the biofilm formed by *K. pneumoniae*, biofilms of all three strains were grown on a polystyrene surface. The non-biofilm former *M. muris* strain 1040/11 (Sager et al., 2015) served as a negative control. For all tested bacteria, 2 × 10^7^ CFU/mL in 50% BHI were incubated in a 96-well plate along with sterile polystyrene platelets (standing vertically in the wells) at 37°C for 24 h in triplicate for each strain. After incubation, the platelets were washed three times by immersion in sterile PBS and fixed with 2% glutaraldehyde and 2% paraformaldehyde solution in 0.1 M PBS for 1 h. Following this, the platelets were washed in PBS 3 × 5 min, incubated in 1% osmium tetroxide solution in distilled water for 1 h, and again washed 3 × 5 min in PBS. The platelets were then dehydrated in a graded series of ethanol concentrations (30, 50, 90%) for 10 min each, followed by three 10-min washes in 99.9% ethanol at room temperature. Subsequently, the platelets were transferred into the Critical Point Dryer (Baltec CPD 030, BAL-TEC GmbH, Schalksmühle, Germany). After critical point drying, the platelets were mounted on aluminum tables, sputter coated with 20 nm gold/palladium (Baltec MED 020, BAL-TEC GmbH, Schalksmühle, Germany), and examined using the SEM secondary electron detector (Zeiss EVO LS 15 LaB6, Carl Zeiss Microscopy Deutschland GmbH, Oberkochen, Germany).

### 2.4 Effect of AMPs on biofilm formation

#### 2.4.1 Determination of planktonic growth above biofilms

The influence of the antimicrobial agents on planktonic growth was determined as described previously (Krieger et al., 2021). Strains were grown overnight in 4 mL BHI broth for approximately 17 h at 37°C on a shaking platform (120 rpm). The overnight culture was then diluted to a McFarland standard of 0.5 in 50% BHI broth and further diluted 1:10 in 50% BHI broth. A serial dilution of the AMPs with concentrations of 0.5–16 μg/mL and erythromycin with concentrations of 1–64 μg/mL in 50% BHI broth was prepared in duplicate in 96-well plates. The standardized bacterial suspension was added to the antimicrobial agent solution in a 1:1 ratio (v:v, 50 μL each) and plates were incubated for 22 h at 37°C under static conditions. Wells containing bacterial suspension only were used as growth controls and wells containing BHI broth and antimicrobial agent solution only were used as blanks. Planktonic growth was measured by optical density at 595 nm (OD_595_) on a BioTek Synergy H1 microplate reader. OD values of the blank were subtracted from all the samples. The experiment was conducted at least six times.

#### 2.4.2 Crystal violet assay for quantification of biofilm formation upon AMP treatment

To determine the effect of the AMPs on biofilm formation of *K. pneumoniae*, a standardized bacterial suspension and serial dilutions of antimicrobial agents were incubated together as conducted for determination of planktonic growth. After incubation the crystal violet assay was performed as described above. The percentage of biofilm formation was calculated based on the growth control. The experiment was conducted at least three times.

#### 2.4.3 Resazurin assay for quantification of metabolic activity

Biofilm viability was quantified using the resazurin assay as previously described (Fontoura et al., 2023) with minor modifications. A standardized bacterial suspension and serial dilutions of antimicrobial agents were incubated together as conducted for determination of planktonic growth and biofilm formation. After incubation, the bacterial suspension was discarded and plates were washed three times with sterile PBS. One hundred microliters of sterile PBS containing 2 μL of resazurin (6.75 mg/mL, in sterile PBS; R7017-1G, Merck KGaA, Darmstadt, Germany) were added per well. After an incubation of 3–5 h at 37°C fluorescence intensity was measured with an excitation wavelength (λ_ex_) of 528 nm and an emission wavelength (λ_em_) of 645 nm by BioTek Synergy H1 microplate reader. Measured values of the blank were subtracted from all the samples. The percentage of metabolic activity was calculated based on the growth control. The experiment was conducted at least three times.

### 2.5 Effect of AMPs on mature biofilms

#### 2.5.1 Fluorescence assay

The proportion of living and dead bacteria was determined using the fluorescence assay FilmTracer™ LIVE/DEAD biofilm viability kit (L10316, Life Technologies GmbH, Darmstadt, Germany). A bacterial count of 2 × 10^7^ CFU/mL was inoculated in 50% BHI broth, 100 μL were added to a 96-well plate in duplicate and plates were incubated for 22 h at 37°C. Wells containing only BHI broth were used as blank. After incubation, the supernatant was discarded and plates were washed three times with sterile PBS. A serial dilution of antibacterial agents in PBS at a concentration range of 2–16 μg/mL for AMPs and 8–64 μg/mL for erythromycin was added to the wells. After incubation for 24 h at 37°C and washing three times with sterile PBS, 50 μL filter-sterilized water containing 0.15 μL each of SYTO™ 9 stain and propidium iodide stain was added to each well. Plates were incubated for 20 min at room temperature in the dark. The staining solution was discarded and the plates were washed three times with sterile PBS. The fluorescence intensity of the SYTO™ 9 stain (λ_ex_ = 470 nm, λ_em_ = 500 nm) and the propidium iodide stain (λ_ex_ = 490 nm, λ_em_ = 635 nm) were measured on a BioTek Synergy H1 microplate reader and corrected for the blank values. The percentage of fluorescence intensity was calculated based on the negative control. The SYTO™ 9/propidium iodide ratio was calculated. The experiment was conducted at least three times.

To test the applicability of SYTO™ 9/propidium iodide staining in the microplate reader, dead and live bacteria of *K. pneumoniae* 17349 were quantified using the CLSM and the fluorescence intensity was measured using the microplate reader for comparison. Biofilm of *K. pneumoniae* 17349 was grown 48 h in a 96-glass bottom well plate, stained with SYTO™ 9 and propidium iodide, incubated 20 min and fluorescence intensity was measured by microplate reader as described for the fluorescence assay. Subsequently, live and dead bacteria were quantified by the confocal laser scanning microscope Leica TCS SP8 (CLSM, Leica Microsystems, Mannheim, Germany) to determine the exact number of SYTO™ 9 or propidium iodide positive cells. Therefore, the CLSM images were deconvoluted (Huygens Professional, version 20.10, SVI, Hilversum, The Netherlands), and then quantified using Imaris software (spot measurement, size 1 μm, quality above 5, Imaris 10.2, Oxford Instruments, Abingdon, UK). After killing with 70% ethanol and an incubation of 45 min, live and dead bacteria were quantified again by CLSM and fluorescence intensity was measured by plate reader as described above.

#### 2.5.2 Confocal laser scanning microscopy with rhodamine B-labeled DJK-5

To illustrate the distribution of an AMP in 48 h-old biofilms of strain 17349, DJK-5 was selected as an example and conjugated with rhodamine B. The distribution was investigated using the confocal laser scanning microscope Leica TCS SP8 (Leica Microsystems, Mannheim, Germany). A bacterial count of 2 × 10^7^ CFU/mL was inoculated in 50% BHI broth, 100 μL was added to a 96-glass bottom well plate (655892, Greiner Bio-One GmbH, Frickenhausen, Germany) in triplicate and incubated for 48 h at 37°C. After incubation, the supernatant was discarded, and 50 μL sterile PBS with 0.15 μL of SYTO™ 9 stain was added to each well and incubated for 20 min at room temperature in the dark. Subsequently, rhodamine B-labeled DJK-5 (RhoB_DJK-5) was added to the wells at a concentration of 8 μg/mL in sterile PBS. SYTO™ 9 was excited at 488 nm (PMT detection range 493–556 nm) and rhodamine B at 561 nm (HyD detection range 565–618 nm). Biofilms were recorded 5 min, 2 h, 7 h, 25 h and 44 h after addition of rhodamine B-labeled DJK-5.

To evaluate if conjugation with rhodamine B influences the antimicrobial effect of DJK-5 on biofilms, two antibiofilm assays were conducted. The effect of the rhodamine B-labeled DJK-5 on mature biofilms of *K. pneumoniae* 17349 in comparison to the unlabeled DJK-5 was investigated using the crystal violet assay in polystyrene 96-well plates and the 96-glass bottom well plate. Twenty two-hours old biofilms were grown in polystyrene 96-well plates and treated with different concentrations of DJK-5 and RhoB_DJK-5 as described for the fluorescence assay. After peptide treatment for 24 h, plates were washed three times with tap water and the crystal violet assay was done as described above to quantify biofilm mass. Measured values of the blank were subtracted from all the samples. The percentage of biofilm formation was calculated based on the growth control. The experiment was conducted four times in duplicates.

Additionally, 48-h old biofilms were grown in a 96-glass bottom well plate in duplicates and treated with DJK-5 or RhoB_DJK-5 (8 and 16 μg/mL) as described for the fluorescence assay. After peptide treatment for 24 h, the well plate was washed with tap water and crystal violet assay was conducted as described above. The percentage of biofilm formation was calculated based on the growth control.

## 3 Results

### 3.1 Biofilm formation of *Klebsiella pneumoniae*

**Figure 1:**
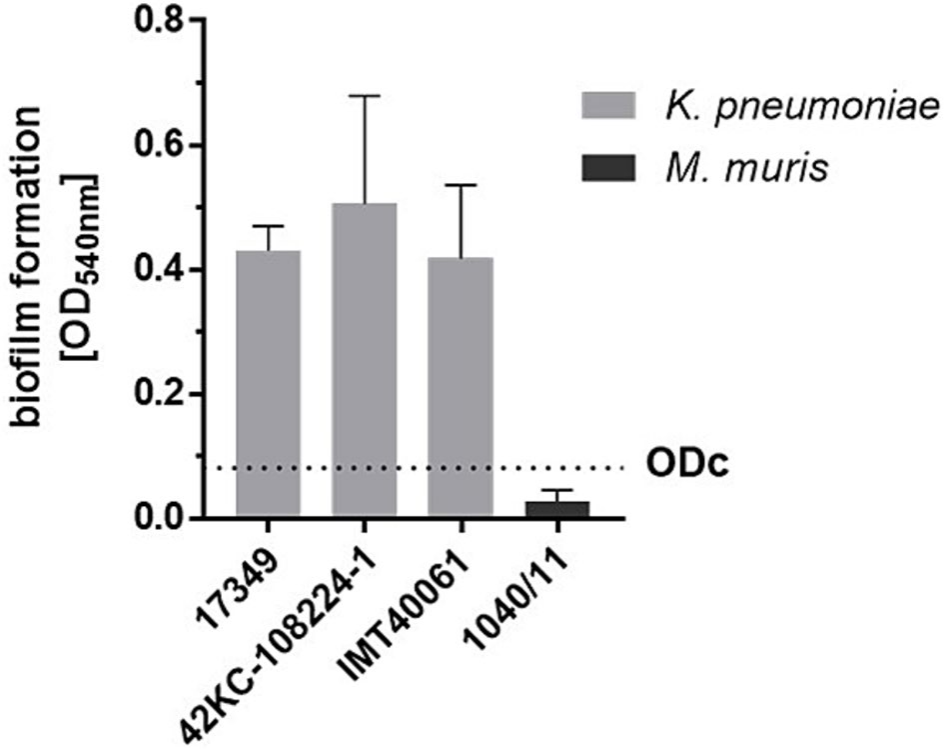
Biofilm formation of a porcine (17349), human (42KC-108224-1) and canine (IMT40061) *K. pneumoniae* strain as determined by the crystal violet assay. A standardized overnight culture of each strain was incubated for 24 h in a 96-well plate. After washing the plate biofilm formation was quantified using the crystal violet assay. The cut-off value (ODc, dashed line) was defined as the mean value of the non-biofilm former *M. muris* plus three times the standard deviation. Bars and error bars represent mean values and standard deviations, respectively (*n* = 3).

Biofilm formation of *K. pneumoniae* on medical devices is associated with severe complications in hospitalized patients (Murphy and Clegg, 2012; Singhai et al., 2012). As *K. pneumoniae* is also a veterinary and zoonotic pathogen, we comparatively investigated inhibition of biofilm formation of three *K. pneumoniae* isolates from different species. First, the crystal violet assay was used to quantify the biofilm mass of the selected strains on polystyrene surfaces. The optical density of all three *K. pneumoniae* strains was 14- to 17-fold higher than the optical density of the non-biofilm former *M. muris* (Figure 1). The human isolate *K. pneumoniae* 42KC-108224-1 had the highest biofilm-forming capacity, with a mean value of 0.51. Biofilm formation of veterinary strains 17349 and IMT40061 was very similar, with mean ODs of 0.43 and 0.42, respectively. In contrast, *M. muris* exhibited a much lower mean optical density of 0.03, resulting in a cut-off value for biofilm formation of 0.08.

**Figure 2:**
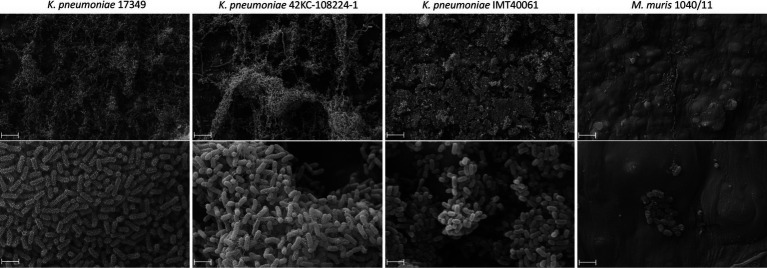
Scanning electron microscopy of biolfilms on polystyrene platelets of a porcine (17349), a human (42KC-108224-1) and a dog (IMT40061) *K. pneumoniae* strain in comparison to the non-biofilm former *Muribacter muris*. Bacterial suspension and polystyrene platelets were incubated together for 24 h before visualization of the formed biofilms with scanning electron microscopy. Scale bars indicate 10 μm (top) and 2 μm (bottom).

For visualization, biofilms of the three *K. pneumoniae* strains grown on polystyrene platelets were examined using SEM (Figure 2). In contrast to *M. muris* 1040/11, the *K. pneumoniae* strains formed dense, multilayered biofilms with a pronounced 3D-structure while only a few single bacteria adhered to the surface of platelets incubated with *M. muris*.

**Figure 3:**
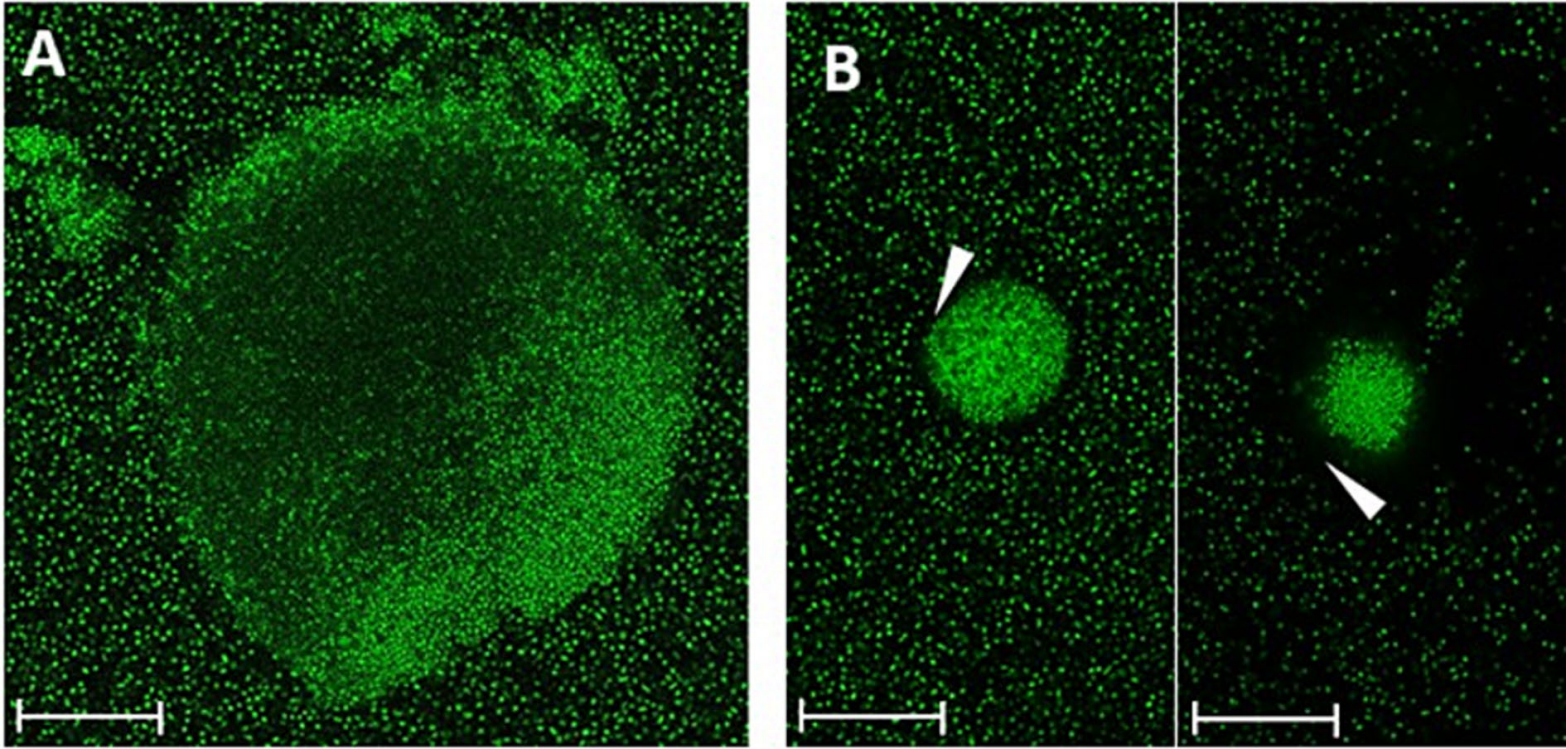
Different morphologies of *K. pneumoniae* strain 17349 biofilms, visualized by CLSM. Biofilms of *K. pneumoniae* were grown for 48 h in a glass bottom 96-well plate and stained with SYTO™ 9, which stains all bacteria fluorescent green. **(A)** *K. pneumoniae* biofilm aggregate with a less dense center. **(B)** Very dense *K. pneumoniae* biofilm aggregates with a cell free zone of about 3 to 12 μm (arrowhead). Scale bars indicate 35 μm.

Different biofilm morphologies of strain 17349 were also observed using CLSM after 48 h of biofilm growth in a glass bottom well plate (Figure 3). There were dense biofilm aggregates with a less dense center (Figure 3A), with most of these aggregates surrounded by a cell-poor area (Figure 3B).

In conclusion, prominent biofilm formation of *K. pneumoniae* strains 42KC-108224-1, 17349, and IMT40061 on polystyrene was detected.

### 3.2 Influence of selected antimicrobial peptides on biofilm formation of *Klebsiella pneumoniae*

**Figure 4:**
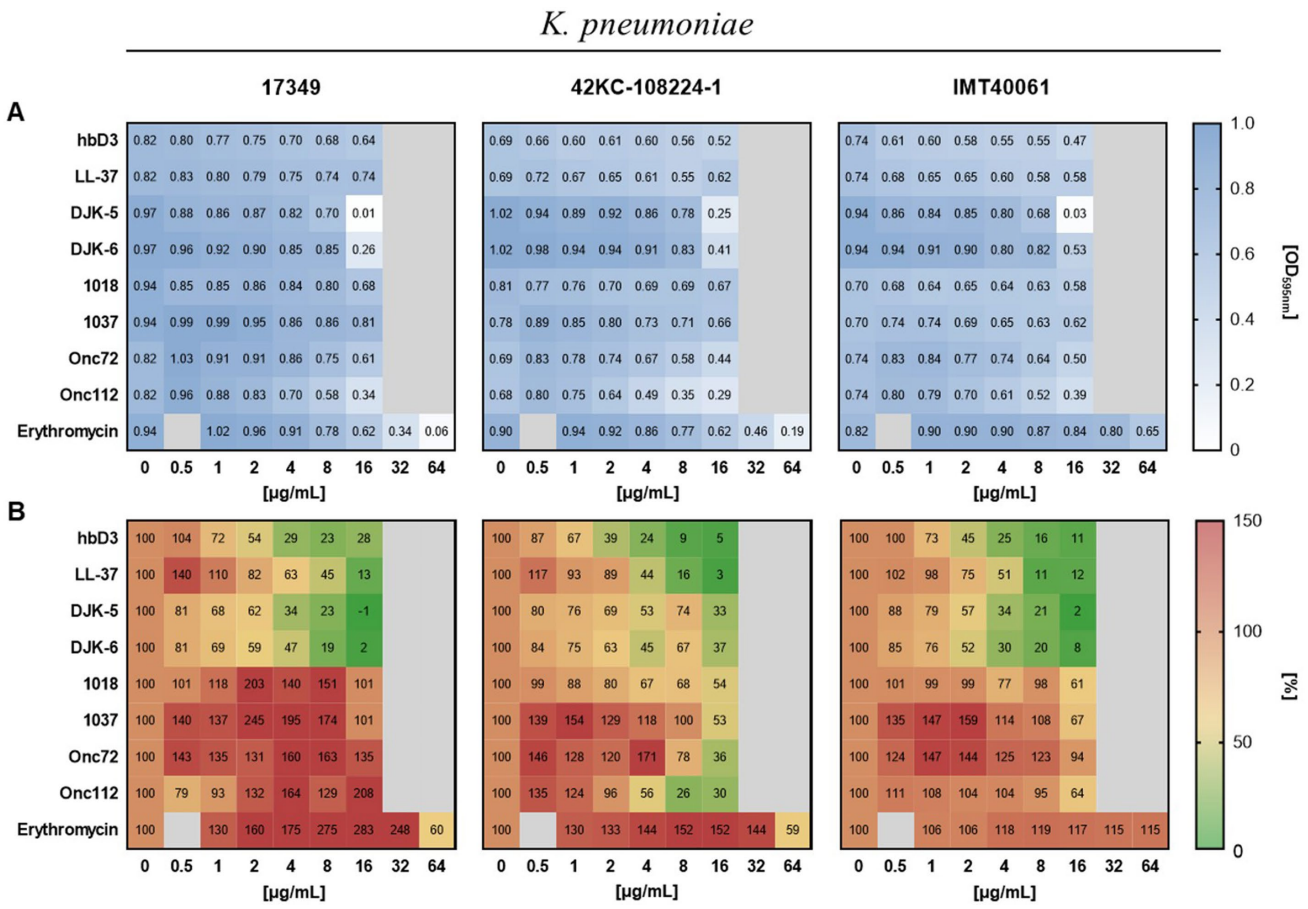
Effect of the indicated AMPs and erythromycin on planktonic growth **(A)** and biofilm formation **(B)** of the different *K. pneumoniae* strains. The standardized bacterial suspension and a serial dilution of AMP or erythromycin were incubated together for 22 h before measuring planktonic growth and biofilm formation. **(A)** Mean values of the optical density at 595 nm indicating planktonic growth (*n* ≥ 6). **(B)** Mean values of biofilm mass quantified by crystal violet assay calculated as percentage of the growth control without AMP treatment (*n* ≥ 3).

We investigated the effect of selected AMPs on *K. pneumoniae* in both forms, planktonic and biofilm-associated. As shown in Figure 4A, only the AMPs DJK-5, DJK-6, Onc72, and Onc112 showed a pronounced effect on the planktonic growth of all three strains.

To determine the effect of AMPs on biofilm formation of *K. pneumoniae*, the biofilm mass remaining in the wells after incubation of bacterial suspensions with AMPs was quantified using crystal violet. The AMPs hbD3, LL-37, DJK-5, and DJK-6 had pronounced inhibitory effect on biofilm formation of all three *K. pneumoniae* strains, reducing biofilm mass to less than 40% of the untreated control, and in some cases, even below 10% (Figure 4B). In contrast to hbD3 and LL-37, the AMPs DJK-5 and DJK-6 also affected planktonic growth at 16 μg/mL, especially in strain 17349. Oncocins Onc72 and Onc112, which had a moderate effect on the planktonic growth of the strains, substantially reduced biofilm mass only for strain 42KC-108224-1, lowering it to 36 and 26% of the untreated control at 16 μg/mL, respectively (Figure 4). The peptides 1018 and 1037 only had little or no effect on either planktonic growth or biofilm formation. However, some peptides even increased biofilm mass at low concentrations. Erythromycin induced an increase in biofilm mass for all three strains at concentrations up to 32 μg/mL but reduced biofilm mass of strains 17349 and 42KC-108224-1 at 64 μg/mL, a concentration that inhibited also planktonic growth. In summary, the selected AMPs exhibited substantial differences, in some cases even opposite effects, on biofilm formation of *K. pneumoniae*, depending on the strain and the concentration.

**Figure 5:**
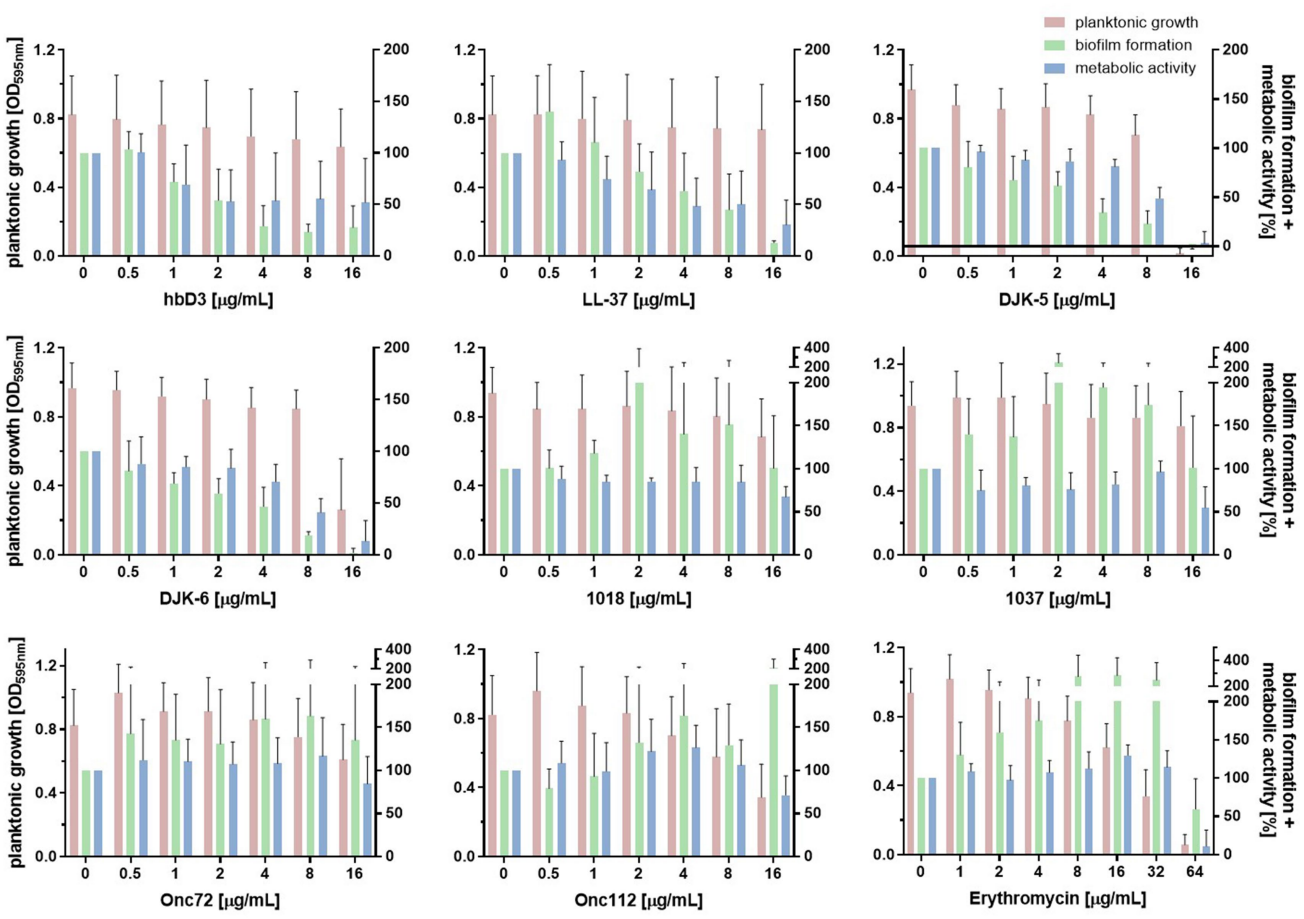
Effect of indicated AMPs and erythromycin on planktonic growth, biofilm formation and metabolic activity of *K. pneumoniae* 17349. After the incubation of a standardized bacterial suspension and the serial dilution of AMP or erythromycin together for 22 h, planktonic growth was estimated measuring the optical density at 595 nm. Either the crystal violet assay was then used to quantify the biofilm mass or the resazurin assay was used to determine the metabolic activity of the biofilm-associated bacteria remaining in the wells. Bars and error bars represent mean values (*n* ≥ 3) and standard deviations, respectively.

Next, we assessed the influence of the AMPs on metabolic activity of the biofilm using the resazurin assay. Resazurin, a weakly fluorescent blue dye, is converted by metabolically active bacteria to resorufin, a highly fluorescent pink product. Figure 5 shows the effect of the investigated AMPs and erythromycin on planktonic growth, biofilm formation and metabolic activity of the porcine *K. pneumoniae* strain 17349 (strains 42KC-108224-1 and IMT40061 shown in Supplementary Figures S2, S3). The AMPs that strongly suppressed biofilm formation in this strain also reduced the metabolic activity of the biofilm-associated bacteria by approximately 50% or more, consistent with substantially decreased viability. For example, at a concentration of 16 μg/mL, LL-37 reduced the biofilm mass to 13% and the metabolic activity to 30%, while planktonic growth was hardly affected. A concentration of 16 μg/mL of DJK-5 or DJK-6 reduced biofilm mass to −1 and 2%, respectively, and the metabolic activity decreased to 3 and 13%, respectively (planktonic growth was also severely suppressed). Unexpectedly, peptide 1037 (16 μg/mL) reduced metabolic activity of the biofilm to 55% while planktonic growth and biofilm mass were hardly affected. At low concentrations, erythromycin led to an increase in the metabolic activity of the biofilm, while a reduction in metabolic activity was observed only at a concentration of 64 μg/mL. Overall, AMPs in general had a comparable effect on the formation and metabolic activity of biofilm-associated bacteria. Among the investigated AMPs, hbD3, LL-37, DJK-5 and DJK-6 (16 or 8 μg/mL) exhibited very pronounced inhibitory effects on the ability of *K. pneumoniae* to form biofilms and on metabolic activity within biofilms.

### 3.3 Influence of various antimicrobial peptides on mature biofilms of *Klebsiella pneumoniae*

Next, we asked whether AMPs would have an effect on the ratio of live to dead bacteria when applied to mature biofilms using a fluorescence assay with the FilmTracer™ LIVE/DEAD biofilm viability kit. The kit contains the fluorescent dye SYTO™ 9, which gives green fluorescence in all bacterial cells while propidium iodide accumulates only in bacteria with damaged membranes causing a reduction in green fluorescence while showing red fluorescence. According to the manufacturer, this could also be used for reading fluorescence in microplate reader (Fisher Scientific, 2025).

**Figure 6:**
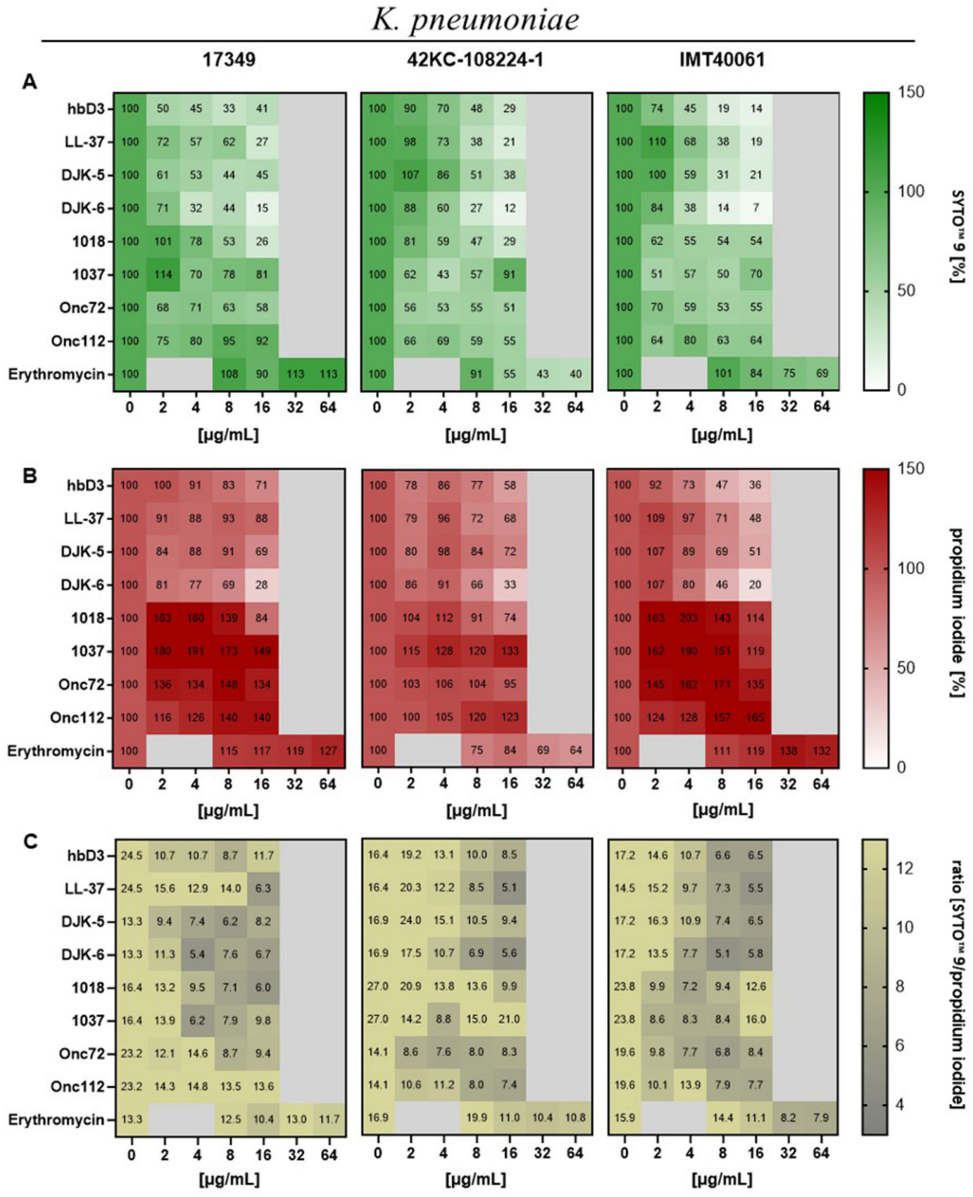
Effect of indicated AMPs and erythromycin on mature biofilms of *K. pneumoniae*. Biofilms were grown for 22 h in 96-well plates before treatment with AMPs or erythromycin for 24 h. After treatment and washing of the plate the remaining biofilm mass was stained with SYTO™ 9 and propidium iodide (FilmTracer™ LIVE/DEAD Biofilm Viability Kit). Fluorescence intensity was measured using the microplate reader. **(A)** Mean values of fluorescence intensity of SYTO™ 9 in percent (*n* ≥ 3), which represents live cells. **(B)** Mean values of fluorescence intensity of propidium iodide in percent (*n* ≥ 3), which represents dead cells. **(C)** Mean values of SYTO™ 9 to propidium iodide.

The data on the fluorescence intensity of SYTO™ 9 is shown in Figure 6A. For all three strains, the 24-h treatment with AMP DJK-6 (16 μg/mL) had the highest effect, reducing the viable biofilm mass to 15% for strain 17349, 12% for strain 42KC-108224-1 and 7% for strain IMT40061. The AMPs hbD3, LL-37 and DJK-5 also showed a strong reduction in live biofilm mass in most cases. Peptide 1018 reduced the viable biofilm mass of strain 17349 and 42KC-108224-1 to less than 30%. Onc72, Onc112 and peptide 1037 were generally less effective to reduce the viable biofilm mass after 24 h of treatment, with the strongest reduction observed in strain 42KC-108224-1, where they reduced viable biofilm mass to 51, 55, and 43%, respectively. The effect of erythromycin was highly strain dependent. For strain 17349, erythromycin caused an increase in living biofilm mass, whereas for strain 42KC-108224-1, it reduced the biofilm mass with increasing concentration. For strain IMT40061, erythromycin treatment also decreased the living biofilm mass, but only to 69% and at very high concentrations.

In Figure 6B, the fluorescence intensity of propidium iodide is shown for different peptide concentrations. Due to the reduction of the whole biofilm mass, the mass of dead bacteria was also clearly reduced by the AMPs hbD3, LL-37, DJK-5, and DJK-6, with the greatest reduction observed under the influence of DJK-6 for all three strains. In contrast, Onc72, Onc112, peptides 1018 and 1037 caused an increase in the number of dead bacteria in the biofilm, except Onc72 and 1018 for strain 42KC-108224-1. Erythromycin caused an increase in fluorescence intensity for strain 17349 and IMT40061, while fluorescence intensity was decreased for strain 42KC-108224-1 to 64% at 64 μg/mL.

The ratio of the fluorescence intensity of SYTO™ 9 to propidium iodide is shown in Figure 6C. As AMP concentration increased, the ratio dropped for all AMPs and strains, so the proportion of dead cells in the biofilm increased.

To test the applicability of SYTO™ 9/propidium iodide staining in the microplate reader, dead and live bacteria of *K. pneumoniae* 17349 were comparatively quantified using the CLSM and measurement of fluorescence intensity in the microplate reader before and after treatment with 70% ethanol. CLSM alaysis confirmed an increase of dead and a decrease of live bacteria after treatment with 70% ethanol. A shift of the SYTO™ 9/propidium iodide ratio from 0.8 to 0.3, as determined by CLSM, was associated with a simultaneous drop from 6.6 to 1.9 of the fluorescence intensity measured in the microplate reader (Supplementary Figure S4).

**Figure 7:**
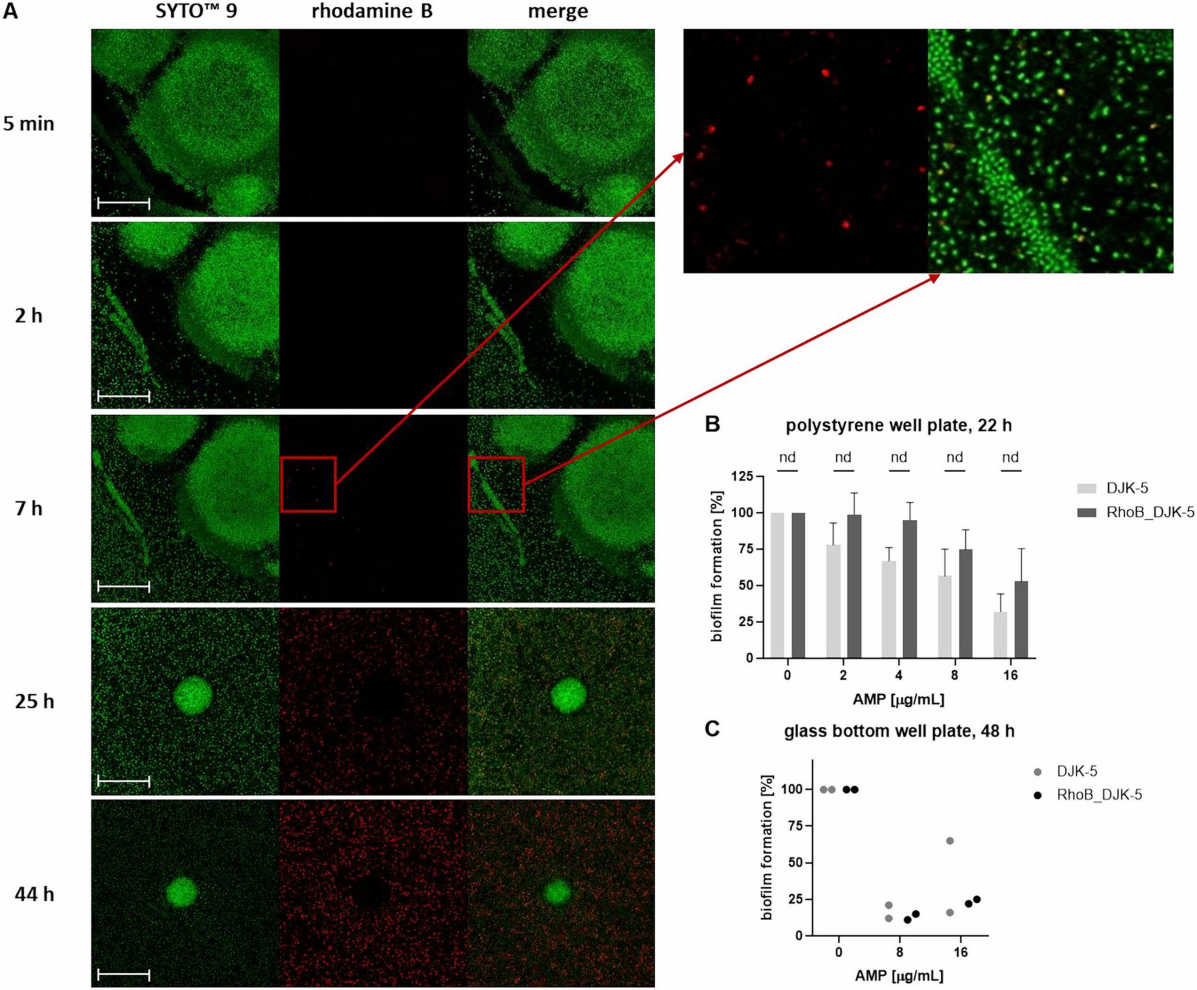
Distribution of rhodamine B-labeled DJK-5 in the biofilm of *K. pneumoniae* strain 17349 **(A)** and comparison of anti-biofilm activity of DJK-5 and RhoB_DJK-5 **(B,C)**. **(A)** Biofilms of *K. pneumoniae* were grown for 48 h in a 96-glass bottom well plate and stained with SYTO™ 9, which stains all bacteria fluorescent green. Rhodamine B-labeled AMP DJK-5 was added to the wells with the mature biofilm, which indicates DJK-5-positive cells fluorescent red. Shown are images of the biofilm at distinct time points after addition of rhodamine B-labeled DJK-5. Scale bars indicate 50 μm. **(B,C)** Crystal violet assay of biofilms of *K. pneumoniae* strain 17349 grown for 22 h in 96-polystyrene plates **(B)** or 48 h in a 96-glass bottom well plate **(C)** after treatment with different concentrations of DJK-5 and RhoB_DJK-5 for 24 h. Bars and error bars represent the mean values of biofilm formation calculated as percentage of the growth control without AMP treatment and standard deviations, respectively **(B)** *n* = 4, **(C)** *n* = 1. Statistical analyses for B were performed using Mann–Whitney test.

Another aim was to visualize the penetration of a potent AMP into the biofilm. For this purpose, DJK-5 was conjugated with the fluorescent dye rhodamine B. We evaluated the effect of conjugation of DJK-5 with rhodamine B on the anti-biofilm activity as follows: Biofilms were grown for 22 h in 96-well plates and 48 h in a 96-glass bottom well plate and treated with different peptide concentrations of conjugated and non-fluorophore labeled DJK-5 (Figures 7B,C). No significant differences in the effect of the two peptides were found using the Mann–Whitney test, though there was a tendency that conjugated DJK-5 reduced the biofilm mass in polystyrene wells less prominently.

The distribution in a 48 h-old biofilm of *K. pneumoniae* strain 17349 was visualized at specific time points during a total treatment time of 44 h. Both live and dead bacteria were stained with the green fluorescent SYTO™ 9. DJK-5-positive bacteria showed a red fluorescence due to rhodamine B labeling of the peptide. As shown in Figure 7A, red fluorescent bacteria were visible for the first 7 h after addition of the AMP. In the course of the following hours, an accumulation of DJK-5 in the planktonic bacteria was detectable, while DJK-5 did not accumulate in the formed biofilm aggregates up to 44 h after addition of the AMP.

In summary, the AMPs hbD3, LL-37, DJK-5 and DJK-6 caused a decrease in fluorescence intensity of both dead and live bacteria, but an increase in the ratio of dead to live bacteria. In contrast, Onc72 and Onc112 specifically increased the fluorescence intensity associated with dead bacteria. The accumulation of rhodamine B-labeled DJK-5 in planktonic bacteria could already be detected after 7 h, while penetration into the dense biofilm aggregates could not be observed.

## 4 Discussion

*K. pneumoniae* is an important pathogen in both human and veterinary medicine, characterized by antimicrobial resistance and biofilm formation (Podschun and Ullmann, 1998; Rahmat Ullah et al., 2024). The threat posed by biofilm-forming pathogens to human health is increasing (Barman et al., 2024), highlighting the urgent need for new antimicrobials, especially biofilm targeting agents. One of the promising alternatives are antimicrobial peptides. The aim of this study was to comparatively evaluate the antimicrobial effect of eight AMPs with different modes of action on planktonic and biofilm-associated bacteria of *K. pneumoniae*. As *K. pneumoniae* is a highly diverse bacterium that causes disease in various animals, we included three unrelated strains originally isolated from different species, which show clear differences in their virulence factors and resistance profiles (Table 2 and Supplementary Table S1). All three strains are able to form biofilms *in vitro*, as demonstrated by crystal violet assay and SEM. Comparative analysis of effects on very different strains provides perspective for identifying both strain independent and strain dependent effects of AMPs. Furthermore, strain 17349 was included to conduct follow-up *in vivo* studies in a porcine model closely resembling humans.

While the effect on biofilms has not yet been investigated for the two PrAMPs Onc72 and Onc112, antibiofilm effects against various Gram-negative and Gram-positive pathogens have already been described for the other AMPs (La Fuente-Núñez et al., 2012; Lee et al., 2013; Zhu et al., 2013; La Fuente-Núñez et al., 2014; La Fuente-Núñez et al., 2015; Chen et al., 2018; Lin et al., 2018; Parducho et al., 2020; Wongkaewkhiaw et al., 2020). However, this is the first study with a systematic comparison of these AMPs in terms of their effect on planktonic growth, biofilm formation, metabolic activity of the biofilm mass and viability of mature biofilms of different *K. pneumoniae* strains. As the biofilm-enhancing effect of erythromycin has already been shown for other pathogens (Wang et al., 2010; Gomes et al., 2013; Cui et al., 2015), erythromycin was included in this study to investigate the effect of sub-MICs on biofilm formation of the three selected strains of *K. pneumoniae*.

Only the AMPs DJK-5, DJK-6, Onc72 and Onc112 inhibited planktonic growth of the three different strains, whereby a narrow concentration range was chosen due to the limited availability of antimicrobial peptides. While DJK-5 led to a complete inhibition of bacterial growth at 16 μg/mL (strains 17349 and IMT40061), the other AMPs only reduced the final OD to 0.26–0.61 at the same concentration, indicating low to medium inhibition. Based on the results of the crystal violet assay, the effect on biofilm formation was highly dependent on the respective AMP, its concentration and the investigated strain. Strain dependence was observed for DJK-5, which at 8 μg/mL was able to reduce biofilm mass of strain 17349 to 23%, but strain 42KC-108224-1 could only be inhibited to 74%. The reason for these differences between strains is not clear, but one might speculate that the thick capsule of the hypervirulent rmpA+ 42KC-108224-1 strain plays a role as it presumably modulates diffusion of AMPs in the cytosol. This might also be a reason for less decreasing dead bacteria after AMP treatment on mature biofilms of strain 42KC-108224-1.

Significant concentration dependent changes were detected in case of LL-37 reducing the biofilm mass of strain 17349 to 13% at 16 μg/mL; however, treatment with 0.5 μg/mL enhanced biofilm formation to 140%. The effects varied significantly between the AMPs. While hbD3, LL-37, DJK-5 and DJK-6 were effective agents against biofilm formation, all other AMPs led to massive increased biofilm mass up to 245%. This comparison confirms that selected AMPs might exert highly distinct anti-biofilm effect at specific concentrations. It should also be noted that some of the peptides differ greatly in size. For example, the AMPs hbD3 and LL-37 are approximately twice as large as the oncocins and four times as large as the short AMPs DJK-5 and DJK-6. A solution with a certain concentration, for example 8 μg/mL, still contains significantly fewer moles of the large AMPs than the small ones, which underlines the strong effect of hbD3 and LL-37 on biofilms of *K. pneumoniae*.

The human defensin hbD3, which reduces biofilm mass to 28% or less, inhibits biofilm formation by influencing the expression of various biofilm-associated genes, for example *icaA*, *icaD*, and *icaR* (Zhu et al., 2013). Even though the effect against biofilms of *K. pneumoniae* has not yet been described, other studies have already shown an antibiofilm effect on Gram-positive bacteria such as *Staphylococcus aureus* and *S. epidermidis* (Zhu et al., 2013) and the Gram-negative pathogen *P. aeruginosa* (Parducho et al., 2020).

The human cathelicidin LL-37 affects bacterial biofilms through different mechanisms of action, for example the reduction of bacterial attachment to the surface, stimulation of twitching motility, quorum sensing pathway suppression and the up- and downregulation of certain biofilm-associated genes (e.g., associated with flagella or type IV pili) (Overhage et al., 2008; Dean et al., 2011; Demirci et al., 2022). A 3 h-treatment with LL-37 inhibits the bacterial attachment to microtiter plates of various clinical isolates of ESKAPE pathogens, including *K. pneumoniae* (Lin et al., 2018). A recent study also showed using the crystal violet assay that incubation with 10 μg/mL of LL-37 for 24 h significantly inhibited biofilm formation of two gentamicin resistant *K. pneumoniae* strains (Chen et al., 2024), which is consistent with our results. The antimicrobial peptides 1018, DJK-5 and DJK-6 presumably affect biofilms by degradation or prevention of accumulation of the signaling nucleotides guanosine 5′-diphosphate 3′-diphosphate (ppGpp) and guanosine 5′-triphosphate 3′-diphosphate (pppGpp), which are induced by stress conditions and are involved in biofilm formation (La Fuente-Núñez et al., 2014; La Fuente-Núñez et al., 2015). The reduced production of ppGpp is possibly a consequence of the downregulation of the expression of SpoT and RelA, enzymes, that metabolise ppGpp (Pletzer et al., 2017). Our findings regarding the ability of the d-enantiomeric peptides DJK-5 and DJK-6 to inhibit biofilm formation of *K. pneumoniae* are also consistent with previous studies, which showed a killing over 98% of the biofilm-associated bacteria in *K. pneumoniae* biofilms, as determined in a BioFlux microfluidic system (La Fuente-Núñez et al., 2015). A study with carbapenemase-producing *K. pneumoniae* also confirmed the good antibiofilm effect of the peptides, that exhibited rather poor efficacy against planktonic bacteria, including peptide 1018 (Ribeiro et al., 2015). La Fuente-Núñez et al. were the first to discover that peptide 1018 was able to prevent *K. pneumoniae* biofilm formation and eradicate 2-days-old biofilms at 2 μg/mL in flow cell chamber experiments. This antibiofilm effect was also observed for other Gram-negative and Gram-positive pathogens (La Fuente-Núñez et al., 2014). These findings differ from our result, as only at a concentration of 16 μg/mL peptide 1018 was able to reduce the biofilm formation of two strains to 61 and 54%, while no antibiofilm effect was detected in strain 17349. However, in an antibiofilm experiment using the crystal violet assay, Jiale et al. were also unable to detect an antibiofilm effect of peptide 1018 up to a concentration of 64 μg/mL against methicillin-resistant *S. aureus* biofilms (Jiale et al., 2021).

La Fuente-Núñez et al. first described the antibiofilm effect of peptide 1037 against biofilms of *P. aeruginosa*, *Listeria monocytogenes* and *Burkholderia cenocepacia* (La Fuente-Núñez et al., 2012). There, peptide 1037 caused a dysregulation of 398 investigated genes (e.g., genes related to flagella, chemotaxis or twitching motility), reduced the swimming and swarming motility and enhanced twitching motility, which may be the reason for biofilm inhibitory effects (La Fuente-Núñez et al., 2012). In our experiments, peptide 1037 was only able to inhibit the biofilms of strain 42KC-108224-1 and IMT40061 to 53 and 67% at a concentration of 16 μg/mL, respectively. At lower concentrations, biofilm formation was even increased in all three strains. The antibiofilm effect of the oncocins Onc72 and Onc112 was strongly dependent on the respective strain. While they led to an increase in biofilm mass in strain 17349, they were able to inhibit biofilm formation in strain 42KC-108224-1 to 36% for Onc72 and 26% for Onc112, with planktonic growth also being suppressed by these AMPs. In addition to the antibacterial effect of oncocins, which has already been described in various models (Knappe et al., 2012; Knappe et al., 2015; Kolano et al., 2020; Krieger et al., 2021, 2022), these AMPs also had a moderate, strain-dependent antibiofilm effect on *K. pneumoniae*. In contrast, erythromycin triggered in all three strains a strong increase in biofilm formation. A reduction in biofilm mass was only achieved at concentrations at which planktonic growth was also inhibited. Moshynets et al. (2022) also observed more pronounced biofilm formation of *K. pneumoniae* under the influence of subinhibitory erythromycin concentrations. Furthermore, biofilm formation of minocycline-resistant *K. pneumoniae* strains is significantly enhanced by subminimum inhibitory concentrations of tetracycline antibiotics (Guo et al., 2024). A possible mechanism of action of erythromycin that leads to increased biofilm formation could be the up- and downregulation of certain bacterial genes, as described for *S. epidermidis* and *Staphylococcus capitis* (Wang et al., 2010; Cui et al., 2015).

Differences between the effect on planktonic bacteria and biofilms can be explained by the different mechanisms of action. While the effect against planktonic bacteria is primarily based on the interaction of the cationic peptides with the negatively charged membrane, the antibiofilm effects of the peptides are based, for example, on the regulation of the expression of certain biofilm-associated genes, influencing bacterial adhesion or quorum sensing or affecting second messenger nucleotides (Luo and Song, 2021). The results also show that the antibiofilm effect on different strains of *K. pneumoniae* can vary widely. Ribeiro et al. also found differences in the antibiofilm effect of the AMPs 1018, DJK-5 and DJK-6 on five different strains of carbapenemase-producing strains of *K. pneumoniae*. There, for example, the minimal concentration required to inhibit biofilm formation by 50% (MBIC_50_) of DJK-5 varies between 32 and 1 μg/mL, depending on the strain (Ribeiro et al., 2015). These differences may be due to the significant variability in the composition of the biofilm matrix, even between different strains of *K. pneumoniae* (Singh et al., 2019). Due to these strain-specific differences, it is particularly important to conduct experiments with strains of different origins.

In order to quantify the metabolic activity of the biofilm mass, the resazurin assay was conducted, in which the resazurin is converted by metabolically active bacteria to the highly fluorescent resorufin. In most cases, the metabolic activity decreased in parallel with the reduction in biofilm mass. These results can be explained by the decrease in the total metabolically active mass, thereby confirming the results of the crystal violet assay. A recent study with comparable results showed that an 18 h-treatment with 1 μM hbD3 on a clinical *P. aeruginosa* strain almost completely inhibited biofilm formation and metabolic activity, as measured in both biofilm-associated and planktonic bacteria (Parducho et al., 2020). However, for some AMPs, the metabolic activity of the biofilm decreased while the biofilm mass was hardly affected. For example, peptide 1037 (16 μg/mL) showed no inhibition of biofilm formation, but reduced metabolic activity to approximately 55% for strain 17349. At these concentrations, the AMPs may reduce the metabolic activity of the bacteria without inhibiting biofilm formation, possibly by influencing genes associated with the metabolic activity of bacteria. La Fuente-Núñez et al. demonstrated the dysregulation of 398 genes after incubation of *P. aeruginosa* strain PAO1 with peptide 1037 using microarray technology, including genes which may play a role in various metabolic pathways of the bacteria (La Fuente-Núñez et al., 2012).

In addition to the effect on biofilm formation, the impact of the AMPs on mature biofilms should also be investigated. Due to the limitations of the crystal violet assay in differentiating between living and dead cells, mature biofilms were stained with SYTO™ 9 and propidium iodide in this part of the study. The four AMPs hbD3, LL-37, DJK-5 and DJK-6 caused a decrease in both living and dead cells, indicating the eradication and dispersal of biofilm mass. However, the proportion of dead cells in the remaining biofilm increased slightly, as the ratio of SYTO™ 9 and propidium iodide dropped with increased peptide concentration. The simultaneous drop of living as well as dead cells can be explained by the detachment of biofilm mass, which consists of dead and living cells (Ma et al., 2009). In a previous study, hbD3 (50 μg/mL) significantly reduced the biovolume of a 21 days-old multispecies biofilm on coverslips after a 24 h-treatment, as determined by CLSM (Lee et al., 2013). The ability of LL-37 to affect mature biofilms has also been demonstrated in previous studies. Specifically, LL-37 showed concentration-dependent killing activity (determined as bacterial survival by plate count technique) on 24 h-old biofilms of *K. pneumoniae*, *P. aeruginosa*, and *S. epidermidis* (Wongkaewkhiaw et al., 2020). Chen et al. also found that an 1 h-treatment with 20 μM LL-37 did not reduce the mass of 24 and 48 h-old *P. aeruginosa* biofilms, but significantly killed viable bacteria in the biofilm, as determined by crystal violet assay, plate count technique, and CLSM (Chen et al., 2018). DJK-5 and DJK-6, at a concentration of only 2.5 μg/mL, were also capable of eradicating 48 h-old *P. aeruginosa* biofilms (La Fuente-Núñez et al., 2015) and Ribeiro et al. demonstrated the ability of DJK-6 (2 μg/mL) to disrupt 48 h-old biofilms of carbapenemase-producing *K. pneumoniae* strains, as determined by a flow cell system (Ribeiro et al., 2015). However, no further studies on the effect of DJK-5 on mature biofilms of *K. pneumoniae* are available to date.

The AMPs Onc72, Onc112, 1018 and 1037 reduced the living biofilm mass, though not as pronounced as with the other AMPs, but was associated with an increase in the dead biofilm mass. In agreement with the results of the crystal violet assay, the total biofilm mass decreased less significantly than with the other AMPs. The results of the resazurin assay also showed that under the influence of the oncocins and peptides 1018 and 1037 the metabolic activity of the biofilm was already partially reduced, while the biofilm mass was hardly affected. PrAMPs, like Onc72 and Onc112, affect the bacterial protein synthesis by binding to the chaperone DnaK (Zahn et al., 2013), but more importantly binding inside the tunnel of the 70S ribosome, blocking the peptidyl transferase center and blocking and destabilization of the initiation complex (Krizsan et al., 2014; Roy et al., 2015; Seefeldt et al., 2015; Gagnon et al., 2016), which could explain the increase in dead bacteria in the biofilm without a decrease in biofilm mass. We hypothesize that the treatment with oncocins and peptides 1018 and 1037 caused less biofilm mass to detach than with the other AMPs (as shown by crystal violet assay), which might be an advantage in cases of increased risks of embolism. Detached biofilm material, which is highly variable in size, is capable of re-adhering and colonizing epithelial cells, as shown by Grønnemose et al. (2017) for *S. aureus* biofilms. However, at the concentrations investigated, there is still a relevant proportion of viable bacteria in the biofilm, so there is still a risk of detachment and spread of these living bacteria when biofilm detaches. The effect of erythromycin was strongly dependent on the strain. While it caused a reduction in the living and dead bacteria of the biofilm in strain 42KC-108224-1, strain 17349 showed an increased number of bacteria even at higher concentrations. A previous study also showed that the ability of erythromycin to eradicate biofilms of *E. coli* and *A. baumannii* was rather moderate and significantly increased when combined with cationic amphiphilic macromolecules (Uppu et al., 2017).

The visualization of biofilms of strain 17349 by CLSM revealed different biofilm morphologies, which may represent various stages of biofilm formation, as observed in *P. aeruginosa* biofilms (Thi et al., 2020). To track the penetration and distribution of the AMP DJK-5, it was conjugated with the fluorescent dye rhodamine B. Surprisingly, the planktonic bacteria were positive for DJK-5 within a few hours, although the AMP was unable to penetrate deeply into the dense biofilm aggregates within the time period investigated. The cell-poor areas visible around the aggregates may represent regions of dense biofilm matrix that prevent the AMP from penetrating. This suggests that DJK-5 efficiently inhibits biofilm formation as long as dense bacterial aggregates have not yet formed. However, we cannot rule out that conjugation of DJK-5 modulates penetration of DJK-5 into dense bacterial aggregates, though we did not record significant differences in the anti-biofilm effect of conjugated and non-conjugated DJK-5. The effect on biofilm formation was confirmed in the experiments using the crystal violet assay. It is possible that these aggregates were washed away in the fluorescence assay carried out to investigate the effect of the AMPs on mature biofilms, and thus were not included in the quantification. In contrast to our results, Wongkaewkhiaw et al. showed, that the d-enantiomer of AMP LL-31 (d-LL-31) was able to rapidly bind to 24 h-old biofilms of *K. pneumoniae*, *S. epidermidis*, and *P. aeruginosa* within an incubation time of only 5 min (Quinn, 2011; Wongkaewkhiaw et al., 2020). However, no comparable studies have yet been carried out for DJK-5.

The biofilm inhibiting and destroying effects of the selected AMPs on *K. pneumoniae* biofilms were very different and, in some cases, strain-dependent. However, there are distinct AMPs, such as hbD3, LL-37, DJK-5 and DJK-6 that have a pronounced effect on biofilm formation and metabolic activity on biofilm-associated bacteria. They are able to reduce the number of both living and dead bacteria in 22 h-old biofilms, making them promising candidates for further studies. However, these findings are currently limited to *in vitro* studies and will need to be expanded to *in vivo* experiments and adapted to clinical situations.

## Data Availability

The datasets presented in this study can be found in online repositories. The names of the repository/repositories and accession number(s) can be found in the article/Supplementary material.

## References

[ref1] Aka S. T. (2015). Killing efficacy and anti-biofilm activity of synthetic human cationic antimicrobial peptide cathelicidin hCAP-18/LL37 against urinary tract pathogens. J. Microbiol. Infect. Dis. 5:168. doi: 10.5799/ahinjs.02.2015.01.0168

[ref2] Anderl J. N. Zahller J. Roe F. Stewart P. S. (2003). Role of nutrient limitation and stationary-phase existence in *Klebsiella pneumoniae* biofilm resistance to ampicillin and ciprofloxacin. Antimicrob. Agents Chemother. 47, 1251–1256. doi: 10.1128/AAC.47.4.1251-1256.2003, PMID: 12654654 PMC152508

[ref3] Barman S. Kurnaz L. B. Leighton R. Hossain M. W. Decho A. W. Tang C. (2024). Intrinsic antimicrobial resistance: molecular biomaterials to combat microbial biofilms and bacterial persisters. Biomaterials 311:122690. doi: 10.1016/j.biomaterials.2024.122690, PMID: 38976935 PMC11298303

[ref4] Bidewell C. A. Williamson S. M. Rogers J. Tang Y. Ellis R. J. Petrovska L.. (2018). Emergence of *Klebsiella pneumoniae* subspecies *pneumoniae* as a cause of septicaemia in pigs in England. PLoS One 13:e0191958. doi: 10.1371/journal.pone.0191958, PMID: 29470491 PMC5823397

[ref5] Bowring B. G. Fahy V. A. Morris A. Collins A. M. (2017). An unusual culprit: *Klebsiella pneumoniae* causing septicaemia outbreaks in neonatal pigs? Vet. Microbiol. 203, 267–270. doi: 10.1016/j.vetmic.2017.03.018, PMID: 28619154

[ref6] Chen H. Wubbolts R. W. Haagsman H. P. Veldhuizen E. J. A. (2018). Inhibition and eradication of *Pseudomonas aeruginosa* biofilms by host defence peptides. Sci. Rep. 8:10446. doi: 10.1038/s41598-018-28842-8, PMID: 29993029 PMC6041282

[ref7] Chen X. Zhang B. He J. Rui X. He T. Zhang L.. (2024). Exploration of antimicrobial peptides in the treatment of gentamicin-resistant *Klebsiella pneumoniae* infection. Infect. Drug Resist. 17, 2591–2605. doi: 10.2147/IDR.S462653, PMID: 38953095 PMC11215974

[ref8] Cui B. Smooker P. M. Rouch D. A. Deighton M. A. (2015). Effects of erythromycin on the phenotypic and genotypic biofilm expression in two clinical *Staphylococcus capitis* subspecies and a functional analysis of Ica proteins in *S. capitis*. J. Med. Microbiol. 64, 591–604. doi: 10.1099/jmm.0.000059, PMID: 25813821

[ref9] De Souza C. M. Da Silva Á. P. Júnior N. G. O. Martínez O. F. Franco O. L. (2022). Peptides as a therapeutic strategy against *Klebsiella pneumoniae*. Trends Pharmacol. Sci. 43, 335–348. doi: 10.1016/j.tips.2021.12.006 35078644

[ref10] Dean S. N. Bishop B. M. van Hoek M. L. (2011). Susceptibility of *Pseudomonas aeruginosa* biofilm to alpha-helical peptides: D-enantiomer of LL-37. Front. Microbiol. 2:128. doi: 10.3389/fmicb.2011.00128, PMID: 21772832 PMC3131519

[ref11] Demirci M. Yigin A. Demir C. (2022). Efficacy of antimicrobial peptide LL-37 against biofilm forming *Staphylococcus aureus* strains obtained from chronic wound infections. Microb. Pathog. 162:105368. doi: 10.1016/j.micpath.2021.105368, PMID: 34942309

[ref12] Fisher Scientific (2025). Invitrogen Filmtracer LIVE/DEAD Biofilm Viability Kit - Cell Analysis Products, Cell Based Assays. Available online at: https://www.fishersci.com/shop/products/molecular-probes-filmtracer-live-dead-biofilm-viability-kit/L10316 (Accessed February 18, 2025).

[ref13] Fontoura I. Veriato T. S. Raniero L. J. Castilho M. L. (2023). Analysis of capped silver nanoparticles combined with imipenem against different susceptibility profiles of *Klebsiella pneumoniae*. Antibiotics 12:535. doi: 10.3390/antibiotics12030535, PMID: 36978403 PMC10044117

[ref14] Gagnon M. G. Roy R. N. Lomakin I. B. Florin T. Mankin A. S. Steitz T. A. (2016). Structures of proline-rich peptides bound to the ribosome reveal a common mechanism of protein synthesis inhibition. Nucleic Acids Res. 44, 2439–2450. doi: 10.1093/nar/gkw018, PMID: 26809677 PMC4797290

[ref15] Gomes D. L. R. Peixoto R. S. Barbosa E. A. B. Napoleão F. Sabbadini P. S. Dos Santos K. R. N.. (2013). SubMICs of penicillin and erythromycin enhance biofilm formation and hydrophobicity of *Corynebacterium diphtheriae* strains. J. Med. Microbiol. 62, 754–760. doi: 10.1099/jmm.0.052373-0, PMID: 23449875

[ref16] Grønnemose R. B. Saederup K. L. Kolmos H. J. Hansen S. W. K. Asferg C. A. Rasmussen K. J.. (2017). A novel in vitro model for haematogenous spreading of *S. aureus* device biofilms demonstrating clumping dispersal as an advantageous dissemination mechanism. Cell. Microbiol. 19:785. doi: 10.1111/cmi.12785 28873268

[ref17] Guerra M. E. S. Destro G. Vieira B. Lima A. S. Ferraz L. F. C. Hakansson A. P.. (2022). *Klebsiella pneumoniae* biofilms and their role in disease pathogenesis. Front. Cell. Infect. Microbiol. 12:877995. doi: 10.3389/fcimb.2022.877995, PMID: 35646720 PMC9132050

[ref18] Guo T. Yang L. Zhou N. Wang Z. Huan C. Zhou J.. (2024). Subminimum inhibitory concentrations tetracycline antibiotics induce biofilm formation in minocycline-resistant *Klebsiella pneumonia* by affecting bacterial physical and chemical properties and associated genes expression. ACS Infect. Dis 10, 2929–2938. doi: 10.1021/acsinfecdis.4c00280, PMID: 38949961 PMC11321339

[ref19] Jiale Z. Jian J. Xinyi T. Haoji X. Xueqin H. Xiao W. (2021). Design of a novel antimicrobial peptide 1018M targeted ppGpp to inhibit MRSA biofilm formation. AMB Express 11:49. doi: 10.1186/s13568-021-01208-6, PMID: 33770266 PMC7997937

[ref20] Knappe D. Adermann K. Hoffmann R. (2015). Oncocin Onc72 is efficacious against antibiotic-susceptible *Klebsiella pneumoniae* ATCC 43816 in a murine thigh infection model. Biopolymers 104, 707–711. doi: 10.1002/bip.22668, PMID: 25968331

[ref21] Knappe D. Fritsche S. Alber G. Köhler G. Hoffmann R. Müller U. (2012). Oncocin derivative Onc72 is highly active against *Escherichia coli* in a systemic septicaemia infection mouse model. J. Antimicrob. Chemother. 67, 2445–2451. doi: 10.1093/jac/dks241, PMID: 22729924

[ref22] Knappe D. Kabankov N. Hoffmann R. (2011a). Bactericidal oncocin derivatives with superior serum stabilities. Int. J. Antimicrob. Agents 37, 166–170. doi: 10.1016/j.ijantimicag.2010.10.028, PMID: 21185160

[ref23] Knappe D. Piantavigna S. Hansen A. Mechler A. Binas A. Nolte O.. (2010). Oncocin (VDKPPYLPRPRPPRRIYNR-NH2): a novel antibacterial peptide optimized against gram-negative human pathogens. J. Med. Chem. 53, 5240–5247. doi: 10.1021/jm100378b, PMID: 20565063

[ref24] Knappe D. Zahn M. Sauer U. Schiffer G. Sträter N. Hoffmann R. (2011b). Rational design of oncocin derivatives with superior protease stabilities and antibacterial activities based on the high-resolution structure of the oncocin-DnaK complex. Chembiochem 12, 874–876. doi: 10.1002/cbic.201000792, PMID: 21387510

[ref25] Kolano L. Knappe D. Volke D. Sträter N. Hoffmann R. (2020). Ribosomal target-binding sites of antimicrobial peptides Api137 and Onc112 are conserved among pathogens indicating new lead structures to develop novel broad-spectrum antibiotics. Chembiochem 21, 2628–2634. doi: 10.1002/cbic.202000109, PMID: 32293093 PMC7540576

[ref26] Krieger A. K. Knappe D. Öhlmann S. Mayer L. Eder I. B. Köller G.. (2021). Proline-rich antimicrobial peptide Api137 is bactericidal in porcine blood infected ex vivo with a porcine or human *Klebsiella pneumoniae* strain. J. Glob. Antimicrob. Resist. 24, 127–135. doi: 10.1016/j.jgar.2020.12.012 33373733

[ref27] Krieger A. K. Öhlmann S. Mayer L. Weiße C. Rieckmann K. Baums C. G. (2022). Porcine iucA+ but rmpA- *Klebsiella pneumoniae* strains proliferate in blood of young piglets but are killed by IgM and complement dependent opsonophagocytosis when these piglets get older. Vet. Microbiol. 266:109361. doi: 10.1016/j.vetmic.2022.109361, PMID: 35131553

[ref28] Krizsan A. Volke D. Weinert S. Sträter N. Knappe D. Hoffmann R. (2014). Insect-derived proline-rich antimicrobial peptides kill bacteria by inhibiting bacterial protein translation at the 70S ribosome. Angew. Chem. Int. Ed. Engl. 53, 12236–12239. doi: 10.1002/anie.201407145, PMID: 25220491

[ref29] La Fuente-Núñez C. Korolik V. Bains M. Nguyen U. Breidenstein E. B. M. Horsman S.. (2012). Inhibition of bacterial biofilm formation and swarming motility by a small synthetic cationic peptide. Antimicrob. Agents Chemother. 56, 2696–2704. doi: 10.1128/AAC.00064-12, PMID: 22354291 PMC3346644

[ref30] La Fuente-Núñez C. Reffuveille F. Haney E. F. Straus S. K. Hancock R. E. W. (2014). Broad-spectrum anti-biofilm peptide that targets a cellular stress response. PLoS Pathog. 10:e1004152. doi: 10.1371/journal.ppat.1004152, PMID: 24852171 PMC4031209

[ref31] La Fuente-Núñez C. Reffuveille F. Mansour S. C. Reckseidler-Zenteno S. L. Hernández D. Brackman G.. (2015). D-enantiomeric peptides that eradicate wild-type and multidrug-resistant biofilms and protect against lethal *Pseudomonas aeruginosa* infections. Chem. Biol. 22, 196–205. doi: 10.1016/j.chembiol.2015.01.002, PMID: 25699603 PMC4362967

[ref32] Lee J.-K. Chang S. W. Perinpanayagam H. Lim S.-M. Park Y.-J. Han S. H.. (2013). Antibacterial efficacy of a human β-defensin-3 peptide on multispecies biofilms. J. Endod. 39, 1625–1629. doi: 10.1016/j.joen.2013.07.035, PMID: 24238461

[ref33] Lin Q. Deslouches B. Montelaro R. C. Di Y. P. (2018). Prevention of ESKAPE pathogen biofilm formation by antimicrobial peptides WLBU2 and LL37. Int. J. Antimicrob. Agents 52, 667–672. doi: 10.1016/j.ijantimicag.2018.04.019, PMID: 29753132 PMC6230315

[ref34] Luo Y. Song Y. (2021). Mechanism of antimicrobial peptides: antimicrobial, anti-inflammatory and antibiofilm activities. Int. J. Mol. Sci. 22:401. doi: 10.3390/ijms222111401, PMID: 34768832 PMC8584040

[ref35] Ma L. Conover M. Lu H. Parsek M. R. Bayles K. Wozniak D. J. (2009). Assembly and development of the *Pseudomonas aeruginosa* biofilm matrix. PLoS Pathog. 5:e1000354. doi: 10.1371/journal.ppat.1000354, PMID: 19325879 PMC2654510

[ref36] Moshynets O. V. Baranovskyi T. P. Cameron S. Iungin O. S. Pokholenko I. Jerdan R.. (2022). Azithromycin possesses biofilm-inhibitory activity and potentiates non-bactericidal colistin methanesulfonate (CMS) and polymyxin B against *Klebsiella pneumonia*. PLoS One 17:e0270983. doi: 10.1371/journal.pone.0270983, PMID: 35776759 PMC9249213

[ref37] Murphy C. N. Clegg S. (2012). *Klebsiella pneumoniae* and type 3 fimbriae: nosocomial infection, regulation and biofilm formation. Future Microbiol. 7, 991–1002. doi: 10.2217/fmb.12.74, PMID: 22913357

[ref38] Overhage J. Campisano A. Bains M. Torfs E. C. W. Rehm B. H. A. Hancock R. E. W. (2008). Human host defense peptide LL-37 prevents bacterial biofilm formation. Infect. Immun. 76, 4176–4182. doi: 10.1128/IAI.00318-08, PMID: 18591225 PMC2519444

[ref39] Parducho K. R. Beadell B. Ybarra T. K. Bush M. Escalera E. Trejos A. T.. (2020). The antimicrobial peptide human beta-defensin 2 inhibits biofilm production of *Pseudomonas aeruginosa* without compromising metabolic activity. Front. Immunol. 11:805. doi: 10.3389/fimmu.2020.00805, PMID: 32457749 PMC7225314

[ref40] Pletzer D. Wolfmeier H. Bains M. Hancock R. E. W. (2017). Synthetic peptides to target stringent response-controlled virulence in a *Pseudomonas aeruginosa* murine cutaneous infection model. Front. Microbiol. 8:1867. doi: 10.3389/fmicb.2017.01867, PMID: 29021784 PMC5623667

[ref41] Podschun R. Ullmann U. (1998). *Klebsiella* spp. as nosocomial pathogens: epidemiology, taxonomy, typing methods, and pathogenicity factors. Clin. Microbiol. Rev. 11, 589–603. doi: 10.1128/CMR.11.4.589, PMID: 9767057 PMC88898

[ref42] Quinn P. J. (2011). Veterinary microbiology and microbial disease. Chicester: John Wiley & Sons Incorporated.

[ref43] Rahmat Ullah S. Jamal M. Rahman A. Andleeb S. (2024). Comprehensive insights into *Klebsiella pneumoniae*: unravelling clinical impact, epidemiological trends and antibiotic-resistance challenges. J. Antimicrob. Chemother. 79, 1484–1492. doi: 10.1093/jac/dkae184, PMID: 38832539

[ref44] Ribeiro M. G. Morais A. B. C. De Alves A. C. Bolaños C. A. D. Paula C. L. De Portilho F. V. R.. (2022). *Klebsiella*-induced infections in domestic species: a case-series study in 697 animals (1997-2019). Braz. J. Microbiol. 53, 455–464. doi: 10.1007/s42770-021-00667-0, PMID: 35018603 PMC8882559

[ref45] Ribeiro S. M. La Fuente-Núñez C. Baquir B. Faria-Junior C. Franco O. L. Hancock R. E. W. (2015). Antibiofilm peptides increase the susceptibility of carbapenemase-producing *Klebsiella pneumoniae* clinical isolates to β-lactam antibiotics. Antimicrob. Agents Chemother. 59, 3906–3912. doi: 10.1128/AAC.00092-15, PMID: 25896694 PMC4468710

[ref46] Rice L. B. (2008). Federal funding for the study of antimicrobial resistance in nosocomial pathogens: no ESKAPE. J. Infect. Dis. 197, 1079–1081. doi: 10.1086/533452, PMID: 18419525

[ref47] Roy R. N. Lomakin I. B. Gagnon M. G. Steitz T. A. (2015). The mechanism of inhibition of protein synthesis by the proline-rich peptide oncocin. Nat. Struct. Mol. Biol. 22, 466–469. doi: 10.1038/nsmb.3031, PMID: 25984972 PMC4456192

[ref48] Sager M. Benten W. P. M. Engelhardt E. Gougoula C. Benga L. (2015). Characterization of biofilm formation in *Pasteurella pneumotropica* and *Actinobacillus muris* isolates of mouse origin. PLoS One 10:e0138778. doi: 10.1371/journal.pone.0138778, PMID: 26430880 PMC4592018

[ref49] Schneider M. Dorn A. (2001). Differential infectivity of two *Pseudomonas* species and the immune response in the milkweed bug, *Oncopeltus fasciatus* (Insecta: Hemiptera). J. Invertebr. Pathol. 78, 135–140. doi: 10.1006/jipa.2001.5054, PMID: 11812116

[ref50] Seefeldt A. C. Nguyen F. Antunes S. Pérébaskine N. Graf M. Arenz S.. (2015). The proline-rich antimicrobial peptide Onc112 inhibits translation by blocking and destabilizing the initiation complex. Nat. Struct. Mol. Biol. 22, 470–475. doi: 10.1038/nsmb.3034, PMID: 25984971

[ref51] Singh A. K. Yadav S. Chauhan B. S. Nandy N. Singh R. Neogi K.. (2019). Classification of clinical isolates of *Klebsiella pneumoniae* based on their *in vitro* biofilm forming capabilities and elucidation of the biofilm matrix chemistry with special reference to the protein content. Front. Microbiol. 10:669. doi: 10.3389/fmicb.2019.00669, PMID: 31019496 PMC6458294

[ref52] Singhai M. Malik A. Shahid M. Malik M. A. Goyal R. (2012). A study on device-related infections with special reference to biofilm production and antibiotic resistance. J. Glob. Infect. Dis. 4, 193–198. doi: 10.4103/0974-777X.103896, PMID: 23326076 PMC3543538

[ref53] Stepanović S. Vuković D. Hola V. Di Bonaventura G. Djukić S. Cirković I.. (2007). Quantification of biofilm in microtiter plates: overview of testing conditions and practical recommendations for assessment of biofilm production by staphylococci. APMIS 115, 891–899. doi: 10.1111/j.1600-0463.2007.apm_630.x, PMID: 17696944

[ref54] Thi M. T. T. Wibowo D. Rehm B. H. A. (2020). *Pseudomonas aeruginosa* biofilms. Int. J. Mol. Sci. 21:671. doi: 10.3390/ijms21228671, PMID: 33212950 PMC7698413

[ref55] Uppu D. S. S. M. Konai M. M. Sarkar P. Samaddar S. Fensterseifer I. C. M. Farias-Junior C.. (2017). Membrane-active macromolecules kill antibiotic-tolerant bacteria and potentiate antibiotics towards gram-negative bacteria. PLoS One 12:e0183263. doi: 10.1371/journal.pone.0183263, PMID: 28837596 PMC5570306

[ref56] Wang Q. Sun F. J. Liu Y. Xiong L.-R. Xie L.-L. Xia P.-Y. (2010). Enhancement of biofilm formation by subinhibitory concentrations of macrolides in icaADBC-positive and -negative clinical isolates of *Staphylococcus epidermidis*. Antimicrob. Agents Chemother. 54, 2707–2711. doi: 10.1128/AAC.01565-09, PMID: 20231401 PMC2876384

[ref57] WHO (2024). *WHO updates list of drug-resistant bacteria most threatening to human health*. Available online at: https://www.who.int/news/item/17-05-2024-who-updates-list-of-drug-resistant-bacteria-most-threatening-to-human-health (Accessed November 21, 2024).

[ref58] Wongkaewkhiaw S. Taweechaisupapong S. Thanaviratananich S. Bolscher J. G. M. Nazmi K. Anutrakunchai C.. (2020). D-LL-31 enhances biofilm-eradicating effect of currently used antibiotics for chronic rhinosinusitis and its immunomodulatory activity on human lung epithelial cells. PLoS One 15:e0243315. doi: 10.1371/journal.pone.0243315, PMID: 33326455 PMC7743948

[ref59] Zahn M. Berthold N. Kieslich B. Knappe D. Hoffmann R. Sträter N. (2013). Structural studies on the forward and reverse binding modes of peptides to the chaperone DnaK. J. Mol. Biol. 425, 2463–2479. doi: 10.1016/j.jmb.2013.03.041 23562829

[ref60] Zhu C. Tan H. Cheng T. Shen H. Shao J. Guo Y.. (2013). Human β-defensin 3 inhibits antibiotic-resistant *Staphylococcus* biofilm formation. J. Surg. Res. 183, 204–213. doi: 10.1016/j.jss.2012.11.048, PMID: 23273885

