# Supplementary material for: Comparative evaluation of antimicrobial peptides: effect on formation, metabolic activity and viability of Klebsiella pneumoniae biofilms
Source: Front Microbiol. 2025 Apr 11;16:1548362. doi: 10.3389/fmicb.2025.1548362 (PMC12021911; doi:10.3389/fmicb.2025.1548362)
Supplement: Supplementary file 1 [file Data_Sheet_1.pdf]

strain	MIC [µg/mL] of indicated antibiotics																	
	Amikacin	Ampicillin	Ampicillin/ Sulbactam	Aztreonam	Cefotaxime	Ceftazidime	Cefuroxime	Ciprofloxacin	Colistin	Doxycycline	Gentamicin	Imipenem	Levofloxacin	Meropenem	Moxifloxacin	Piperacillin	Piperacillin/ Tazobactam	Tobramycin
17349	<0.5	16	1/4	<0.25	<0.0625	<0.25	1	<0.03125	0.25	0.5	0.25	<0.125	<0.0625	<0.125	0.25	2	1/4	<0.125
42KC-108224-1	<0.5	>32	8/4	<0.25	<0.0625	<0.25	2	0.0625	0.25	1	<0.125	<0.125	<0.0625	<0.125	0.0625	4	1/4	0.25
IMT40061	4	>32	>32/4	>16	>8	>32	>32	>4	<0.0625	4	0.5	<0.125	>8	<0.125	>4	>64	>64/4	4

Table S1 Minimum inhibitory concentration (MIC) values of the three *K. pneumoniae* strains, determined according to EUCAST guidelines by microdilution test.

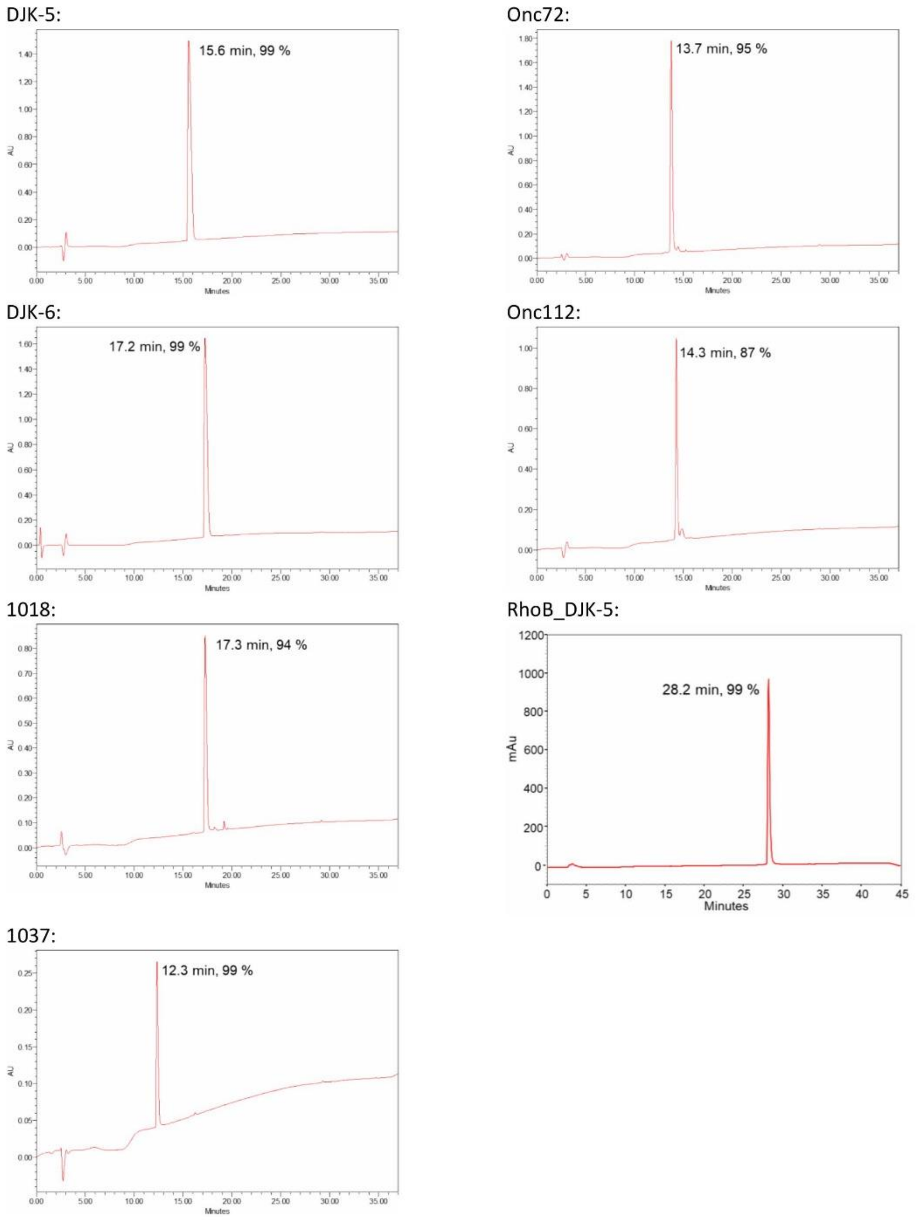

Figure S1 RP Chromatograms of synthetic peptides. Peptides were analyzed by RP-HPLC on a Jupiter C18- column using a linear 28.5-min gradient from 3% to 60% acetonitrile containing 0.1% formic acid (DJK-5, DJK-6, Onc72, Onc112, 1018, 1037) or a linear 30-min gradient from 5% to 95% aqueous acetonitrile containing 0.1% TFA (RhoB_DJK-5). Separations were performed at a column temperature of 60 °C and the absorbance was recorded at 214 nm. Retention times and purities are indicated.

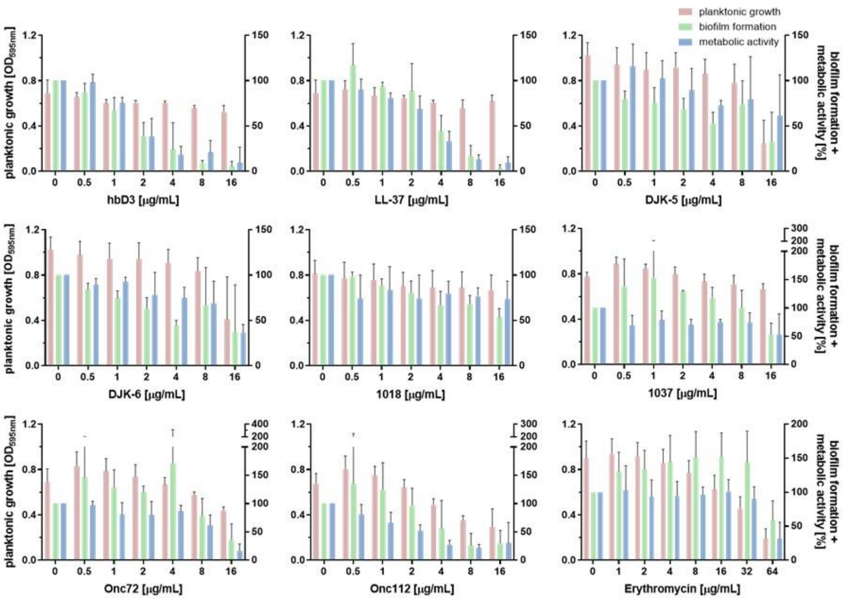

Figure S2 Effect of indicated AMPs and erythromycin on planktonic growth, biofilm formation and metabolic activity of *K. pneumoniae* 42KC-108224-1.

After the incubation of a standardized bacterial suspension and the serial dilution of AMP or erythromycin together for 22 h, planktonic growth was estimated measuring the optical density at 595 nm. Either the crystal violet assay was then used to quantify the biofilm mass or the resazurin assay was used to determine the metabolic activity of the biofilm-associated bacteria remaining in the wells. Bars and error bars represent mean values (n ≥ 3) and standard deviations, respectively.

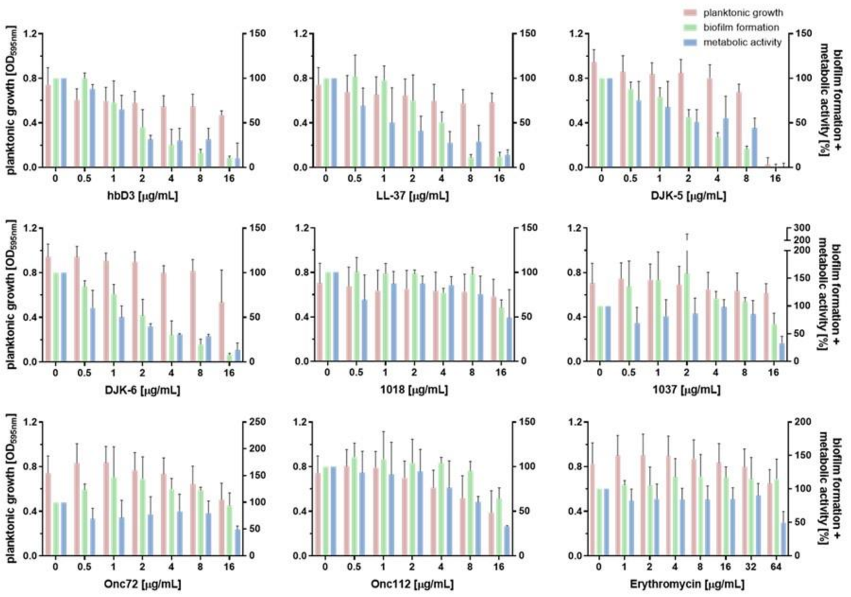

Figure S3 Effect of indicated AMPs and erythromycin on planktonic growth, biofilm formation and metabolic activity of *K. pneumoniae* IMT40061.

After the incubation of a standardized bacterial suspension and the serial dilution of AMP or erythromycin together for 22 h, planktonic growth was estimated measuring the optical density at 595 nm. Either the crystal violet assay was then used to quantify the biofilm mass or the resazurin assay was used to determine the metabolic activity of the biofilm-associated bacteria remaining in the wells. Bars and error bars represent mean values ($n \geq 3$) and standard deviations, respectively.

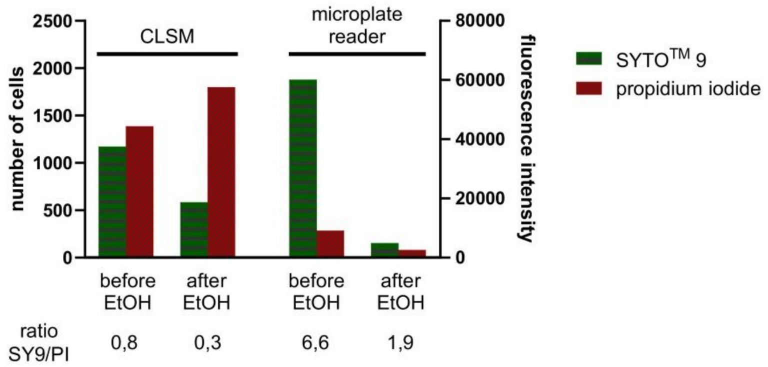

Figure S4 Quantification of live and dead cells by CLSM in comparison to fluorescence intensity determined by microplate reader of 48-h old biofilms stained with SYTO™ 9 and propidium iodide before and after treatment with ethanol (EtOH). Biofilm of *K. pneumoniae* 17349 was grown for 48h in a 96-glass bottom well plate and stained with SYTO™ 9 and propidium iodide. The ratio of SYTO™ 9 (SY9) to propidium iodide (PI) was calculated before and after incubating with 70% ethanol for 45 minutes.
